# CanID: A Robust and Accurate RNA-seq Expression-based Diagnostic Classification Scheme for Pediatric Malignancies

**DOI:** 10.1093/gpbjnl/qzaf122

**Published:** 2025-11-29

**Authors:** Daniel K Putnam, Alexander M Gout, Delaram Rahbarinia, Meiling Jin, David Finkelstein, Xiaotu Ma, Jinghui Zhang, David A Wheeler, Larissa V Furtado, Xiang Chen

**Affiliations:** St Jude Children’s Research Hospital, Department of Computational Biology, Memphis, TN 38105, USA; St Jude Children’s Research Hospital, Department of Computational Biology, Memphis, TN 38105, USA; St Jude Children’s Research Hospital, Department of Computational Biology, Memphis, TN 38105, USA; St Jude Children’s Research Hospital, Department of Computational Biology, Memphis, TN 38105, USA; St Jude Children’s Research Hospital, Department of Computational Biology, Memphis, TN 38105, USA; St Jude Children’s Research Hospital, Department of Computational Biology, Memphis, TN 38105, USA; St Jude Children’s Research Hospital, Department of Computational Biology, Memphis, TN 38105, USA; St Jude Children’s Research Hospital, Department of Computational Biology, Memphis, TN 38105, USA; St Jude Children’s Research Hospital, Department of Pathology, Memphis, TN 38105, USA; St Jude Children’s Research Hospital, Department of Computational Biology, Memphis, TN 38105, USA

**Keywords:** Cancer classification, Machine learning, RNA sequencing, Solid tumor, Hematologic malignancy

## Abstract

Cancer subtype classification is critical for precision therapy, and there is a growing trend to augment histopathology testing with omics-based machine learning classifiers. However, analytical challenges remain in pediatric cancer regarding the scope and precision of current classifiers, as well as the evolving subtype standardization. To address these challenges, we constructed Cancer Identification (CanID), a stacked ensemble machine learning classification scheme, using transcriptomic features derived from gene-level RNA sequencing count data as the sole input. CanID was developed primarily from 3203 pediatric cancer samples across 13 solid tumor subtypes and 38 hematologic malignancy subtypes, with subtype labels curated without the use of RNA-seq data. The accuracies of independent testing in three independent or external datasets for solid tumors and hematologic malignancies were 99% and 92%–93%, respectively. Notably, CanID was able to classify subtypes challenging for clinical histology evaluation and was robust to both biological and technical challenges, including differences in data collection protocols, class imbalance, potential mislabeled training samples, and classes unobserved during training. The high accuracy, robustness, and biological interpretability of this transcriptome-based classification scheme represent a valuable approach to advance tumor diagnosis and clinically meaningful stratification of tumor types. CanID can be accessed on GitHub at https://github.com/chenlab-sj/CanID.

## Graphical Abstract

**Graphical Abstract:**
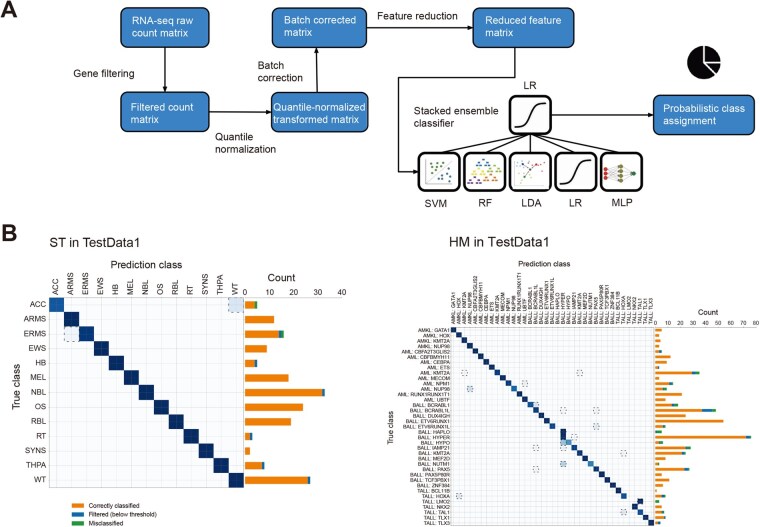

## Introduction

Despite advances in therapy and patient care, pediatric cancer remains the leading cause of death among children, with over 16,000 new cases each year in the United States [1] and 400,000 globally [2]. Subtype classification provides critical input for risk stratification, which determines treatment intensity and eligibility for targeted therapy [3,4] with the ultimate goal of developing more effective and less toxic therapies for pediatric cancer patients. Traditionally, this has been achieved through multiple tests using biomarker-based molecular diagnoses and histopathological approaches. These methods are constrained by the effort required for assay development and validation, as well as the need for iterative testing. As genome-wide profiling has become a standard practice in pediatric oncology testing, subtype classification is increasingly performed by applying validated computational analyses to genome-wide omics data, rather than designing assays for individual biomarkers. For example, fusion gene expression [5] and multi-gene expression signature analyses [6,7] have been incorporated into routine clinical diagnosis for specific cancers. Moreover, DNA methylome-based classifiers have been developed for accurate tumor subtyping in brain tumors [8] and for early tumor detection in cell-free DNA [9].

The high accuracy achieved by methylation-based classifiers, which were developed to analyze pediatric central nervous system (CNS) tumors using the random forest (RF) algorithm, has established the feasibility of deriving an unbiased machine learning-based subtype classification scheme. A limitation of the DNA methylation platform is the lack of direct functional interpretability of the underlying signal measured by methylation probes. In contrast, features extracted from gene-based whole transcriptome sequencing (*i.e.*, RNA-seq) analyses have the potential to generate a biologically interpretable classification scheme. RNA-seq is also a key genomic data type generated by major pediatric cancer genomic research initiatives, *e.g.*, St. Jude / Washington University Pediatric Cancer Genome Project (PCGP [10]) and the National Cancer Institute (NCI) Therapeutically Applicable Research to Generate Effective Treatments (TARGET [11]) as well as clinical sequencing programs (*e.g.*, Genomes for Kids (Genome4Kids) [12], Individualized Therapy for Relapsed Malignancies (INFORM) [13], and Zero Childhood Cancer Precision Medicine Program (ZERO) [14]). This enables the assembly of a sufficient number of RNA-seq samples from public databases, which is essential for training and validating classifiers for very rare pediatric cancer subtypes. When combined with methods for visualizing high-dimensional data, such as t-distributed stochastic neighbor embedding (tSNE), RNA-seq has proven useful for classifying tumors in research settings [15–17]. Leveraging RNA-seq data in this way is particularly useful for discerning tumor subtypes without relying on known biomarkers.

Machine learning approaches are well-suited for classification tasks, as they are designed to learn a mapping between a set of observations (*e.g.*, input omics features) and an outcome (*e.g.*, cancer subtypes) from an existing reference dataset (training set). If the learned mapping is generalizable, it can be applied to classify new query samples. Although the classification performance of a machine learning algorithm is typically measured by numerical metrics such as prediction accuracy, precision, recall, F1 score, area under the receiver operating characteristic curve (AU-ROC), and confusion matrices, in many real-world applications, numerical metrics alone often fail to capture a complete description [18]. In addition to achieving high accuracy, it is often desirable for well-performing machine learning models to reveal how a certain classification (or decision) is made, especially in misclassified cases.

Recently, multiple machine learning classifiers for pediatric cancers using RNA-seq have been developed [19–21], providing subtype classification independent of biomarker-based assays and potentially streamlining molecular diagnosis and narrowing the scope of histopathological testing. However, to date, these approaches have either been limited in their scope of B-cell acute lymphoblastic leukemia (B-ALL) cases [20], restricted to certain classifiable subtypes of B-ALL [21], or applicable only to samples prepared using the Poly(A)-enriched mRNA-seq protocol [19].

Here, we report the development of Cancer Identification (CanID), a unified classification scheme designed to classify pediatric tumors using diverse RNA-seq data. CanID normalizes systematic differences across the major RNA-seq protocols, corrects potential batch effects between sample cohorts, and extracts biologically important features using principal component analysis (PCA). To predict the type of individual tumor transcriptomes, we built a stacked ensemble classifier from five base classifiers. To demonstrate the utility of this approach, we focused on pediatric non-CNS solid tumors (STs) and hematologic malignancies (HMs) due to the lack of well-accepted molecular classifiers, and we compared its performance with that of the recently published pediatric tumor classifier, OTTER [19]. OTTER’s developers trained the model using an ensemble of convolutional neural networks on whole-transcriptome gene expression data from poly(A) RNA-seq samples. They identified OTTER classes using a hierarchical clustering algorithm, RACOON.

We built CanID models and classified approximately 4800 samples consisting of 13 subtypes of pediatric STs and 38 subtypes of pediatric HMs, including acute myeloid leukemia (AML), B-ALL, and T-cell acute lymphoblastic leukemia (T-ALL), profiled using mRNA-seq or total RNA-seq [22]. We compiled these samples from seven research initiatives, two clinical sequencing programs, and two external data sources (Tables S1–S5). Users can run CanID as a standalone method or integrate it into a workflow that incorporates ancillary biomarker information, such as gene fusion or somatic mutations, which can also be computed from RNA-seq data.

## Method

### RNA-seq datasets and subtype designation

We obtained all RNA-seq data from public resources including (1) 3202 samples profiled by 7 research studies and 2 clinical sequencing initiatives from St. Jude Cloud (SJCloud) [23] (https://www.stjude.cloud/); (2) 1555 samples from the NCI’s Therapeutically Applicable Research to Generate Effective Treatments initiative [11] (TARGET; https://ocg.cancer.gov/programs/target/data-matrix); (3) 8 rhabdoid tumor (RT) samples from Australian’s The Zero Childhood Cancer program [14] for the purpose of expanding the sample size of RT used for training and testing; and (4) 61 samples from a clinical pilot study [24]. Details of these samples are summarized in Tables S1–S4: summary of the training, testing, and other groups used in CanID development (Table S1); distribution of tumor samples across cohort source (Table S2), classification performance of CanID across independent test datasets (Table S3), and prediction outcomes for rare cases across cohorts (Table S4).

We obtained subtype information of the SJCloud clinical samples from clinical diagnosis guided by the World Health Organization (WHO) criteria [25], key molecular findings, and input from St. Jude pathologists with domain expertise in the relevant tumor types. For research samples, we derived subtype information primarily from the corresponding publications [10,12,16,26–33] (Table S5). Because both the clinical and research samples were collected over a 10-year period, some diagnostic criteria may have changed. To ensure consistency in diagnostic labeling, we harmonized diagnostic subtype annotations for tumors in this study by re-reviewing all cases, examining clinical notes from patients’ electronic medical records, confirming the presence of diagnostic biomarkers when appropriate, and consulting with pathologists who specialize in hematologic and STs. In some cases, this process led to subtype reclassification. For example, several samples labeled as T-lineage ALL of HOXA (TALLHOXA) subtype in a recent publication [16] were reclassified as acute myeloid leukemia with re-arranged KMT2A (AMLKMT2A) based on a review of the original publication [34] and analysis of their gene expression profiles: SJMLL021, SJMLL022, SJMLL012, SJMLL011, SJMLL013, SJMLL018, SJMLL020. Similarly, sample SJMLL030007, also labeled TALLHOXA in the same publication [16], was labeled AMLNPM1 based on gene fusion status reported in a different study [24]. Subtype designations for the TARGET cohort were based in part on the Genomic Data Commons (https://portal.gdc.cancer.gov) and from published literature [16,35].

### RNA-seq data processing

We measured gene expression levels using RNA-seq feature counts. For RNA-seq data hosted on SJCloud, we obtained the corresponding “Feature Count” files from the SJCloud Genomic Platform (https://platform.stjude.cloud/). We computed RNA-seq feature counts for TARGET and ZERO using the same SJCloud pipeline (https://stjudecloud.github.io/rfcs/0001-rnaseq-workflow-v2.0.0.html) [23]. Briefly, we mapped RNA-seq reads to the hg38 genome build (GRCh38_no_alt) with the STAR aligner in two-pass mode [36] and annotated them with GENCODE v31 gene models (https://www.gencodegenes.org/human/release_31.html). We then generated raw gene-level counts with HTSeq-count [37], also using GENCODE v31 gene models.

### Filtering protocol-sensitive genes

To reduce protocol-dependent variation and ensure compatibility across library preparation methods [poly(A) *vs*. total RNA], we applied a multi-step gene filtering procedure: (1) Low-expression filtering: We excluded genes with a maximum counts-per-million (CPM) value below 5 across all samples. (2) Ranking and normalization: We ranked expression values for each gene and converted them to percentiles within each protocol group. (3) Detection of protocol-sensitive genes: We excluded genes if the median percentile shifted by at least one quartile (≥ 25 percentile points) between protocols. To assess distributional overlap, we calculated the 5th and 95th percentile expression values for each gene within each protocol-defined sample class. We excluded genes if the 95th percentile in one protocol was lower than the 5th percentile in the other, indicating a lack of overlap. We applied this criterion in both directions [poly(A) *vs*. total RNA]. Additionally, we removed genes if 95% of expression values in one protocol consistently exceeded — or fell below — 95% of values in the other protocol.

To further ensure compatibility with earlier genome builds and improve annotation reliability, we excluded 1283 genes present only in GENCODE v31 and removed all histone genes. After applying all filters and restricting the dataset to protein-coding genes, we retained a final set of 17,061 genes for downstream analysis.

### Quantile normalization and batch correction

We trained a quantile normalization model on ST (*n* = 456) and leukemia (*n* = 1313) training samples. To prepare the data, we added a value of 1 to the dataset and converted the matrix to the log_2_ scale. We then applied the trained model to the entire data matrix. Next, we computed the mean across samples and saved the rank ordering of the means. We performed quantile normalization using the qnorm package in Python, which took the raw data matrix and the saved rank-ordered means as input and returned the quantile-normalized matrix.

Using the quantile-normalized matrix, we performed frozen surrogate variable analysis (fSVA) after training an SVA model on the training samples [38]. fSVA extends standard SVA by removing unwanted variation from new samples using adjustment factors learned from the training dataset. We first applied SVA to the training set to identify surrogate variables that represent latent factors capturing batch effects. The algorithm then “froze” the relationship between these surrogate variables and the expression features by storing the projection matrix. For each new sample, fSVA used this projection to estimate surrogate variables, regressed them out of the sample’s expression profile, and produced variation-corrected data directly comparable to the training set.

### PCA and feature selection

We assessed the discriminatory power of principal components (PCs) in differentiating tumor classes by performing PCA on the solid-tumor training set. This analysis generated multiple feature sets, each capturing different proportions of variance (PCA65, PCA70, PCA75, PCA80, PCA85, PCA90, PCA95, and PCA99). For each dataset, we applied one-way analysis of variance (ANOVA) to the PC scores, grouping samples by their corresponding ST types. We calculated the resulting *F*-statistics and associated *P* values for each PC to quantify its ability to distinguish between tumor classes and adjusted the *P* values for multiple testing using the Benjamini–Hochberg false discovery rate (FDR) correction.

We ranked the PCs according to their adjusted *P* values and found that PCs 1–9 consistently showed the greatest discriminatory power across all feature sets (Table S6). Pairwise scatter plots of the top 9 PCs demonstrated clear class separation, with distinct clustering patterns observed across tumor types (Figure S1).

### Bootstrap and OBB error estimation for optimal feature selection

We performed bootstrap aggregation by creating two independent sets. The bootstrap set contained the “in-the-bag” data by sampling with replacement, and the number of samples matched the size of the training sets for the ST (*n* = 456) and HM (*n* = 1313) datasets. The out-of-bag (OOB) set contained all data not selected during sampling. We generated 300 independent bootstrapped sets, each consisting of bootstrap samples and the corresponding OOB samples, from the quantile normalized batch corrected (QNBC) matrix. For each dataset, we performed PCA on the QNBC matrix to capture 65% (solid only), 70% (solid only), 75%, 80%, 85%, 90%, 95%, and 99% of the variance explained. Additionally, we generated a feature set of the top 1000 most variable genes from the training datasets for ST and HM separately. We trained our models using the bootstrapped samples and evaluated performance by running the corresponding OOB samples through each trained classifier. We ultimately used the PCA feature set that explained 80% of the variance for STs and 85% of the variance for HMs (Figure S2; Table S7).

### Probability threshold determination from out-of-bag testing

We computed an overall threshold to retain 95% of ST samples and 90% of HM samples from the OOB training results. When a class contained too few samples to meet these retention levels, we set the threshold cutoff to the maximum of 0.5 and the minimum threshold observed across all samples in any minor class.

### Stacking classifier

We enhanced the predictive performance by implementing a stacking ensemble classifier with the StackingClassifier from scikit-learn [39] (v1.1.1) in Python3 (v3.10.4). We selected this approach because it leverages the complementary strengths of diverse classifiers, often improving generalization performance compared to individual models. In this ensemble, we combined multiple base learners (level 0 models) and used their predictions as input features to train a final estimator (level 1 model). The final estimator learned how to optimally combine the outputs of the base learners to produce the final classification. To prevent information leakage, the StackingClassifier trained the base learners in parallel on the full training data and generated inputs for the final estimator using cross-validated predictions. This internal cross-validation ensured that each training prediction for the meta-model came from a fold where the base learner had not been trained on the corresponding sample.

In our implementation, we used linear discriminant analysis (LDA), support vector machine (SVM), multilayer perceptron (MLP), multi-class logistic regression (LR), and RF as base learners. We selected these models to represent a diverse range of machine learning paradigms. LR was chosen as the final estimator because of its simplicity and robustness in combining probabilistic outputs. The SVM was calibrated using scikit-learn’s CalibratedClassifierCV with cross-validation to improve the reliability of the predicted probabilities. This calibration ensured that the probabilistic outputs fed into the final estimator were well calibrated, producing more accurate and interpretable confidence scores in the final predictions.

### Rationale for model selection

We selected five base learners to ensure a diverse representation of machine learning paradigms, with each model contributing complementary strengths. LDA provides a simple and interpretable linear model that performs well when the class distributions are approximately Gaussian. SVM offers a powerful margin-based classifier that handles high-dimensional data and captures non-linear boundaries through kernel functions. MLP serves as a flexible function approximator capable of capturing complex, non-linear relationships in the data. Multi-class LR delivers a probabilistic, linear approach to classification with strong performance on linearly separable data. RF provides ensemble-based, tree models that capture non-linearities and feature interactions while maintaining robustness against overfitting.

We chose this combination to leverage the distinct inductive biases of each model, thereby enabling the stacking ensemble to capture both simple and complex decision boundaries and improve the generalization performance. The stacked ensemble produced a final class prediction with a calibrated confidence score. Table S8 lists the specific parameters used in each base classifier.

### Gene set enrichment analysis

We used ranked gene set enrichment analysis (GSEA, v4.3.3) to identify biological processes associated with the weighted loadings from PCA. We performed the analysis using the biological process set c5.go.bp.v2025.1.Hs.symbols.gmt.

### Identification of PC features that distinguish sample groups

We identified PCA features that discriminate between two tumor types by performing the Student’s *t*-test on each PCA feature and comparing training samples from tumor type 1 with those from tumor type 2. We adjusted the resulting *P* values for multiple hypothesis testing using the Benjamini–Hochberg procedure. We selected PCA features with a FDR *q* < 1 × 10^−20^. If no features met this threshold, we applied a relaxed criterion of FDR *q* < 1 × 10^−15^ (Table S9).

### Fusion transcript detection with Fuzzion2 pattern-matching algorithm

We used Fuzzion2 v1.4.0 [40] to detect gene fusions. Detecting gene fusions is critical in discovering cancer drivers and for clinical oncology testing; however, most existing software tools require hours to run and often miss lowly expressed fusions. Fuzzion2 applies pattern matching to identify known gene fusions in unmapped paired-read RNA-seq data. Given a set of patterns representing fusion transcript breakpoints, it finds every read pair that matches at least one of the patterns. It detects both exact and inexact (fuzzy) matches, with fuzzy matching tolerating variations caused by sequencing errors, single nucleotide variants (SNVs), and indels. By using a novel index of frequency minimizers, Fuzzion2 processes a sample in a few minutes.

### RNA-seq reprocessing pipeline for cross-alignment classifier evaluation

We evaluated the robustness of classifier performance with respect to differences in genome build, annotation version, and aligner, by selecting 25 samples with RNA-seq reads aligned using both BWA to the hg19 reference genome (GENCODE v19) and STAR (v2.0) to the hg38 reference genome (GENCODE v31). For the BWA hg19 GENCODE v19 alignments, we sorted BAM files and extracted paired-end FASTQ reads, trimmed adapter sequences, and realigned the reads with STAR in a two-pass mode to both hg19 (GENCODE v19) and hg38 (GENCODE v31) reference builds.

We quantified gene-level expression using HTSeq-count with the GENCODE annotation corresponding to the genome build used for alignment (v19 for hg19, v31 for hg38). We then processed the resulting HTSeq count matrices from each alignment strategy using the trained CanID model. For each method, we recorded accuracy, number of scored samples, number of correct predictions, and filtered counts (predictions below the classification threshold) to assess the impact of genome build, annotation version, and aligner on classification performance.

## Results

### Overview of CanID prediction pipeline and data

#### RNA-seq preprocessing and stacked ensemble classification workflow

**Figure 1:**
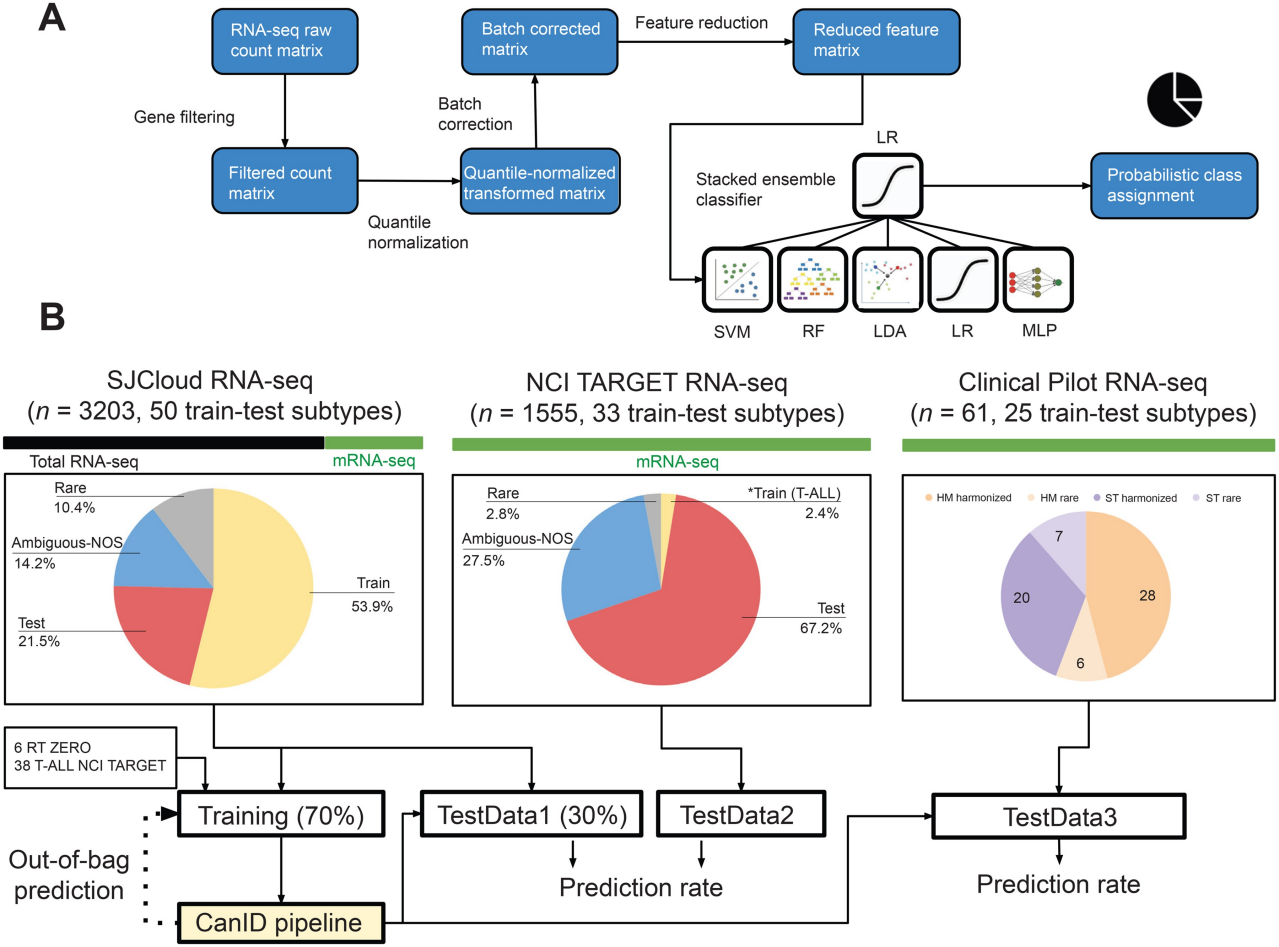
Overview of the CanID pipeline and evaluation strategy **A**. CanID pipeline: Input RNA-seq data undergo an initial gene filtering step to reduce differences between total RNA and poly(A) mRNA capture protocols. The filtered data are then processed through three sequential transformations: (1) quantile normalization, (2) fSVA batch correction, and (3) PCA for feature reduction. A stacked ensemble classifier is subsequently trained on the reduced PCA feature set. **B**. Testing strategy: CanID was evaluated across three independent test sets. TestData1 consists of approximately 30% of SJCloud patient samples (held out from a 70/30 train-test split). To ensure representation of rare subtypes, six RT samples were included in the training set, while two additional RT samples from the ZERO project were included in TestData1. In addition, 38 T-ALL; samples from the NCI TARGET, applicable research to generate effective treatment; initiative were included in the training set (BCL11B = 4, NKX2 = 11, TAL2 = 6, TLX1 = 17), and 13 were held out in TestData1 (BCL11B = 1, NKX2 = 4, TAL2 = 1, TLX1 = 7). TestData2 is an independent external TARGET dataset containing 1555 samples across 33 subtypes. TestData3 comprises an independent set of clinical pilot samples. CanID, Cancer Identification; SJCloud, St. Jude Cloud; NCI, National Cancer Institute; TARGET, Therapeutically Applicable Research to Generate Effective Treatments; PCA, principal component analysis; RT, rhabdoid tumor; ZERO, zero childhood cancer precision medicine program; T-ALL, T-cell acute lymphoblastic leukemia; RNA-seq, RNA sequencing; LDA, linear discriminant analysis; RF, random forest; SVM, support vector machine; MLP, multilayer perceptron; LR, multi-class logistic regression; ST, solid tumor; HM, hematologic malignancie; fSVA, frozen surrogate variable analysis; NCI TARGET, National Cancer Institute’s therapeutically.

Our classification scheme, CanID, uses RNA-seq feature count data for protein-coding genes (see Method). We used the feature count matrix from the training data as the raw input and processed it sequentially through (1) quantile normalization, (2) batch correction using frozen surrogate variable analysis (fSVA) [38], and (3) PCA for feature reduction. We trained a stacked ensemble model for cancer subtype classification using information-dense PCA features (**Figure 1**A). In the prediction phase, the trained stacked ensemble model takes the read count data for each test sample as input, transforms them using a set of frozen preprocessing models learned in the training phase, and outputs the predicted class along with a confidence score.

#### Cohorts and RNA-seq data sources for model development

As shown in Figure 1B, we used three major cohorts: 3202 RNA-seq samples (935 ST and 2267 HM samples) obtained from the SJCloud [23] platform; 1555 RNA-seq samples (464 ST and 1091 HM samples) profiled by the TARGET [11] initiative, and 61 samples (27 ST and 34 HM samples) obtained from a Clinical Pilot cohort that supported the development of clinical cancer genomic profiling platforms at St. Jude Children’s Research Hospital [24]. For the SJCloud cohort, we obtained subtype information for samples profiled by clinical groups from clinical diagnoses and for samples profiled by research groups from research classifications. We harmonized and curated these samples across the entire cohort, as they were collected over a 10-year period during which some diagnostic criteria may have changed (see Method). For the TARGET cohort, we derived subtype information from the TARGET data matrix or published literature. For the Clinical Pilot project, we obtained subtype information from detailed clinical records and molecular testing results.

The SJCloud cohort included both total RNA-seq (approximately 75%) and mRNA-seq (approximately 25%) samples profiled by seven research and two clinical initiatives (Table S5). The TARGET cohort contained mRNA-seq samples exclusively. The Clinical Pilot cohort represented a separate clinical initiative comprising exclusively of total RNA-seq samples.

##### Assembly and curation of training and validation cohorts

We assembled the training and validation datasets primarily from the SJCloud cohort. To enable model development for RT samples and several rare T-ALL subtypes, we added 8 RT samples from the Zero Childhood Cancer project [14] and 51 T-ALL cases from the TARGET cohort: BCL11B (*n* = 5), NKX2 (*n* = 15), TAL2 (*n* = 7), and TLX1 (*n* = 24) (**Tables 1** and **2**, Table S10).

**Table 1:** ST disease counts

Disease	Name	SJ-S Total	SJ-S Train	SJ Test1	SJ-P Total	SJ-P Train	SJ-P Test1	TGT-S Test2	TGT-S Train	ZERO-S Test1
ACC	Adrenocortical carcinoma	19	14	5	19	14	5	0	0	0
ARMS	Alveolar rhabdomyosarcoma	40	28	12	38	27	11	0	0	0
ERMS	Embryonal Rhabdomyosarcoma	52	36	16	45	32	13	0	0	0
EWS	Ewing sarcoma	35	26	9	33	24	9	0	0	0
HB	Hepatoblastoma	18	13	5	16	12	4	0	0	0
MEL	Melanoma	62	44	18	52	37	15	0	0	0
NBL	Neuroblastoma	101	68	33	87	61	26	168	0	0
OS	Osteosarcoma	85	61	24	70	49	21	88	0	0
RBL	Retinoblastoma	68	49	19	64	45	19	0	0	0
RT	Rhabdoid tumor	6	5	1	6	5	1	65	6	2
SYNS	Synovial sarcoma	11	9	2	8	6	2	0	0	0
THPA	Thyroid papillary carcinoma	30	22	8	29	21	8	0	0	0
WT	Wilms tumor	102	75	27	76	54	22	130	0	0
	Total	629	450	179	543	387	156	451	6	2

*Note:* **SJ,** St. Jude Cloud; **S, s**ample; **P**, patient; **TGT,** therapeutically applicable research to generate effective treatment (TARGET); Test1, TestData1; Test2, TestData2; ZERO, zero childhood cancer precision medicine program; ST, solid tumor.

**Table 2:** HM disease counts

Disease	SJ-S Total	SJ-S Train	SJ Test1	SJ-P Total	SJ-P Train	SJ-P Test1	TGT-S Total	TGT-S Train	TGT-S Test1	TGT-S Test2
AMKL: GATA1	19	14	5	19	14	5	0	0	0	0
AMKL: HOX	13	10	3	13	10	3	0	0	0	0
AMKL: KMT2A	19	15	4	16	12	4	0	0	0	0
AMKL: NUP98	10	7	3	10	7	3	0	0	0	0
AML: CBFA2T3GLIS2	18	13	5	18	13	5	5	0	0	5
AML: CBFBMYH11	42	30	12	42	30	12	46	0	0	46
AML: CEBPA	30	21	9	30	21	9	10	0	0	10
AML: ETS	13	10	3	12	9	3	7	0	0	7
AML: KMT2A	125	90	35	118	83	35	51	0	0	51
AML: MECOM	12	9	3	12	9	3	0	0	0	0
AML: NPM1	48	34	14	48	34	14	13	0	0	13
AML: NUP98	33	24	9	31	22	9	19	0	0	19
AML: RUNX1RUNX1T1	77	56	21	69	49	20	41	0	0	41
AML: UBTF	30	22	8	29	21	8	2	0	0	2
BALL: BCRABL1	58	40	18	57	40	17	5	0	0	5
BALL: BCRABL1L	163	115	48	162	114	48	39	0	0	39
BALL: DUX4IGH	82	58	24	82	58	24	6	0	0	6
BALL: ETV6RUNX1	187	133	54	180	126	54	21	0	0	21
BALL: ETV6RUNX1L	29	21	8	28	20	8	5	0	0	5
BALL: HAPLO	17	12	5	17	12	5	0	0	0	0
BALL: HYPER	256	180	76	256	180	76	45	0	0	45
BALL: HYPO	23	17	6	23	17	6	0	0	0	0
BALL: IAMP21	94	66	28	94	66	28	17	0	0	17
BALL: KMT2A	94	70	24	79	56	23	3	0	0	3
BALL: MEF2D	27	19	8	27	19	8	5	0	0	5
BALL: NUTM1	12	9	3	12	9	3	0	0	0	0
BALL: PAX5	90	63	27	90	63	27	19	0	0	19
BALL: PAX5P80R	18	13	5	18	13	5	0	0	0	0
BALL: TCF3PBX1	38	27	11	38	27	11	27	0	0	27
BALL: ZNF384	20	14	6	20	14	6	15	0	0	15
TALL: BCL11B	2	2	0	2	2	0	5	4	1	0
TALL: HOXA	27	19	8	27	19	8	33	0	0	33
TALL: LMO2	11	8	3	9	7	2	13	0	0	13
TALL: NKX2	5	4	1	5	4	1	15	11	4	0
TALL: TAL1	22	15	7	21	15	6	86	0	0	86
TALL: TAL2	0	0	0	0	0	0	7	6	1	0
TALL: TLX1	6	5	1	6	5	1	24	17	7	0
TALL: TLX3	14	10	4	14	10	4	48	0	0	48
Total	1784	1275	509	1734	1230	504	632	38	13	581

*Note:* HM, hematologic malignancy; Train, training set.

We excluded the following categories of samples from the model training: (1) 367 samples (16 ST and 351 HM samples) categorized as ambiguous or not otherwise specified (ambiguous-NOS, Table S11) — a designation for samples that could not be definitively classified into any known disease subtypes, (2) 36 ST samples categorized as rhabdomyosarcoma (RMS), NOS–RMS samples that could not be further subclassified (Table S12), (3) 52 ST samples that we considered to have low confidence after pathologist review (Table S13) and 334 samples (202 ST and 132 HM samples) that belonged to rare diagnostic categories with too few samples ( < 10) to build a classifier (Table S14). To avoid potential information leakage, we placed multiple samples from the same patient in either the training or validation set but did not split them across both groups. We used qualified samples from 70% of the patients for training, which included 456 ST (13 subtypes) and 1313 HM samples [AML (14 subtypes), B-ALL (16 subtypes), and T-ALL (8 subtypes)]. We reserved samples from the remaining 30% of the patients for validation (TestData1), including 181 ST and 522 HM samples (Tables 1 and 2, Table S15).

##### Independent external and clinical testing datasets

TestData2 contains the TARGET cohort using samples not included in the training and serves as an independent validation cohort (Tables 1 and 2). We assigned 451 ST and 581 HM samples to TestData2. We used this dataset to test the generalizability of the classifier, given that the TARGET data are an external dataset (Table S16). TestData3 contains the Clinical Genomics Pilot cohort with 20 harmonized ST samples (8 subtypes), and 28 harmonized HM samples (17 subtypes). We assigned one sample, SJAML030025_D1, to the rare category owing to its ambiguous subtype annotation (Table S17). We assigned the remaining samples from the Clinical Genomics Pilot cohort to the rare group (7 ST and 6 HM samples; Table S18). We included this pan-cancer clinical set to provide an independent comparison between the results of CanID and OTTER because (1) these samples were not observed in the training phase of either pipeline and (2) they have extensive clinical records and molecular testing results that allow us to derive disease subtypes objectively.

### Model construction and testing

#### Optimized features for ST and HM datasets

Given the significant difference between the ST and HM samples, we built separate models for the ST and HM datasets. PCA is a widely used dimensionality reduction algorithm that transforms potentially correlated features into a smaller set of orthogonal PC vectors. By reducing data dimensionality while retaining important information, PCA supports the construction of a robust classifier (see Method). Therefore, we applied PCA to the training data and transformed the input gene array (approximately 17,000 genes) into a smaller set of information-rich features used as inputs for our classification model.

To optimize feature selection, we first tested various numbers of features based on the variance explained. For example, PCA75 represents the feature set generated using PCA to explain 75% of the variance in the input data. Additionally, we generated a set of the top 1000 most variable genes from the input genes in the training datasets. We used bootstrapping to identify the optimal number of PCs to include in the PCA model (Table S7). Briefly, for each feature set generated at a given percentage of explained level, we trained 10 individual CanID models with bootstrapped training sets and repeated the process across 30 batches. We combined the OOB classification accuracy with the model complexity (number of features retained) to select the PCA feature sets that explained 80% of the variance for ST and 85% for HM, which balanced both the prediction accuracy and model complexity (**Table 3**; Figure S2A). We observed that PCA95 and PCA99 showed a significant drop in performance compared to the other PCA sets used for the HM dataset, indicating that they captured the noise in the training data (Figure S2B and C).

**Table 3:** Feature set accuracy in ST and HM samples

Train Dataset	Feature set1	Feature set2	Mean ACC set1	STDEV set1	Mean ACC set2	STDEV set2	Paired *t*-statistic	*P* value	FDR-adjusted *P* value
ST	PCA65	PCA80	0.9763	0.0017	0.9840	0.0021	−7.3335	0.0000	0.0002
ST	PCA70	PCA80	0.9818	0.0015	0.9840	0.0021	−2.3717	0.0418	0.0654
ST	PCA75	PCA80	0.9838	0.0021	0.9840	0.0021	−0.2641	0.7976	0.8302
ST	PCA80	PCA85	0.9840	0.0021	0.9735	0.0032	8.6678	0.0000	0.0001
ST	PCA80	PCA90	0.9840	0.0021	0.9761	0.0046	6.0000	0.0002	0.0007
ST	PCA80	PCA95	0.9840	0.0021	0.9831	0.0046	0.6882	0.5086	0.5722
ST	PCA80	PCA99	0.9840	0.0021	0.9728	0.0067	5.2509	0.0005	0.0015
ST	PCA80	TOP1000	0.9840	0.0021	0.9846	0.0021	−0.7579	0.4679	0.5433
HM	PCA75	PCA85	0.9449	0.0022	0.9506	0.0021	−5.7998	0.0003	0.0004
HM	PCA80	PCA85	0.9491	0.0022	0.9506	0.0021	−1.5519	0.1551	0.1714
HM	PCA85	PCA90	0.9506	0.0021	0.9487	0.0040	2.2165	0.0539	0.0666
HM	PCA85	PCA95	0.9506	0.0021	0.7452	0.0080	89.1276	0.0000	0.0000
HM	PCA85	PCA99	0.9506	0.0021	0.5550	0.0074	154.4059	0.0000	0.0000
HM	PCA85	TOP1000	0.9506	0.0021	0.9453	0.0030	4.4447	0.0016	0.0024

*Note*: For the ST feature sets, PCA80 and TOP1000 achieved the highest average accuracies. PCA80 had no significant difference between PCA PCA70, PCA75, PCA95, or TOP1000. In contrast, PCA80 significantly outperformed PCA65, PCA85, PCA90, and PCA99 at an FDR-adjusted significance threshold of 0.05. Based on this balance between model parsimony and predictive accuracy, we selected PCA80 as the optimal feature set. For the HM feature sets, PCA85 achieved the highest average accuracy, with no significant difference compared to PCA80 or PCA90. In contrast, PCA85 significantly outperformed PCA75, PCA95, PCA99, and TOP1000 at an FDR-adjusted significance threshold of 0.05. PCA85 was therefore selected as the optimal feature set for HM. PCA80, principal component feature set that accounted for 80% of the variances; TOP1000, the feature set containing the top 1000 variable genes.

The PCA feature reduction procedure produced a relatively small set of information-enriched features. The selected PCA feature sets — PCA80 in ST (capturing 80% of the variance, 69 features) and PCA85 in HM (capturing 85% of the variance, 340 features) — demonstrated great differences among tumor types (Figure S3) and captured biological functions essential to these tumors (Figure S4). For example, GSEA [41,42] applied to the gene loadings for PC2 from the ST classifier revealed enrichment of immune-related signatures, including pathways associated with the adaptive immune response on the negative leading edge, and pathways related to mitotic regulation and myogenic development on the positive leading edge (Figure S4; Table S19). Consequently, tumors originating from developing skeletal muscle (*i.e.*, alveolar rhabdomyosarcomas, or ARMS, and embryonal rhabdomyosarcoma, or ERMS) showed higher PC2 values, whereas tumors arising from high immune cell infiltration [*i.e.*, thyroid papillary carcinoma (THPA)] had large negative PC2 values (Figure S3). Similarly, PC1 captured neurotransmitter transport and retina function on the positive leading edge and bone development on the negative leading edge (Figure S4; Table S20). Consistent with the biological processes identified, retinoblastoma (RBL) and neuroblastoma (NBL) exhibited the highest PC1 values, while osteosarcoma (OS) samples showed lower negative PC1 values (Figure S3).

#### Classifier architecture, confidence thresholding, and performance evaluation

CanID uses an ensemble of five individual base classifiers: LDA, SVM, MLP, LR, and RF (see details in Method). Another logistic regression model integrates the individual base-classifier outputs to produce the final prediction and confidence score. We developed a confidence threshold for the final prediction using the OOB predictions from all 300 bootstrapped models combined. CanID classified any predictions with a value below the threshold and excluded them from the valid results. We calculated the confidence score to ensure that approximately 95% (ST) and 90% (HM) of the samples were above the threshold for each tumor type.

**Figure 2:**
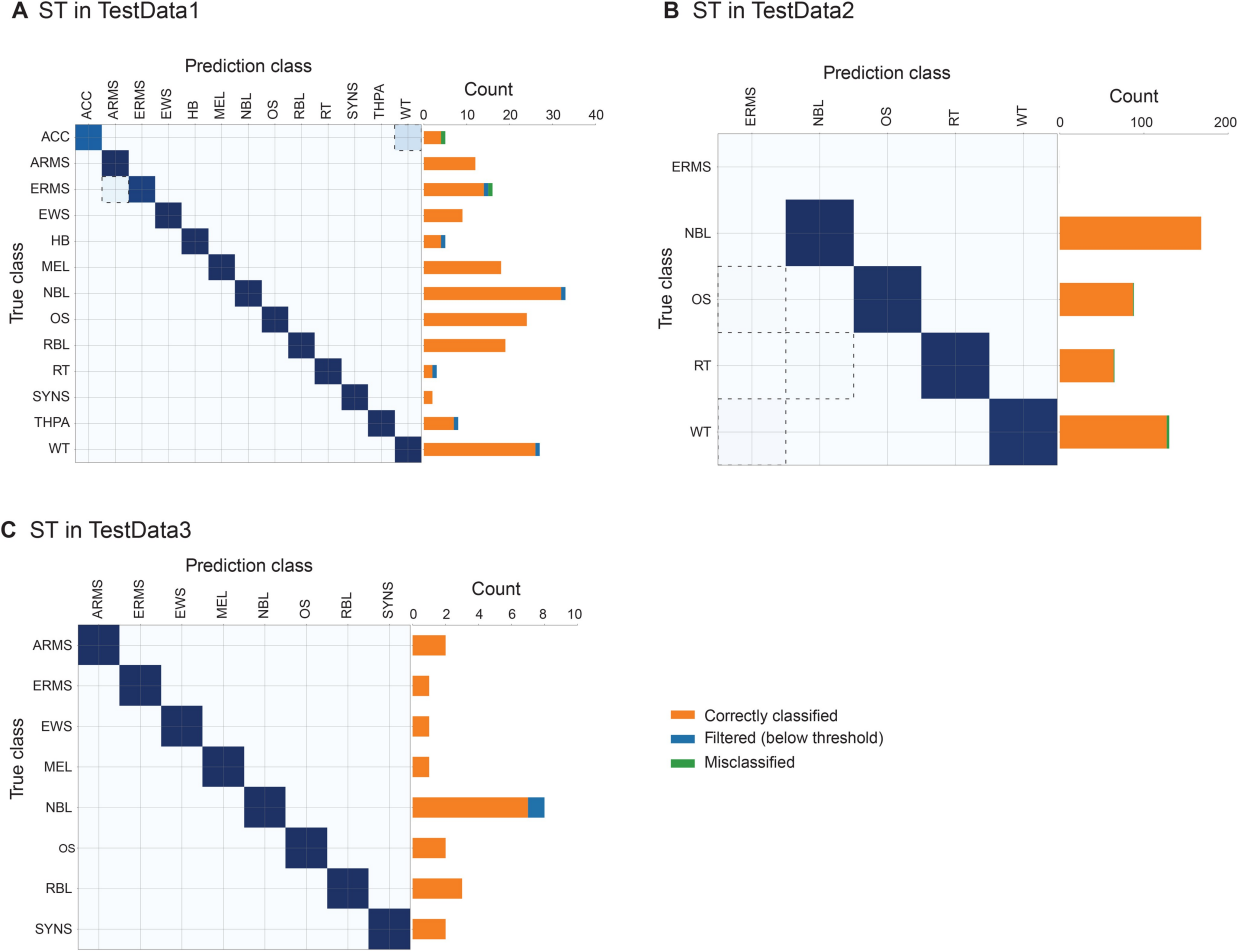
Performance of CanID on ST test sets TestData1 consisted of 30% reserved SJCloud samples supplemented with 2 RT cases from the ZERO project and 13 T-ALL cases from NCI TARGET (BCL11B = 1, NKX2 = 4, TAL2 = 1, TLX1 = 7). Dashed boxes highlight misclassified sample pairs. The accompanying bar chart shows the distribution of filtered (blue), correctly classified (orange), and misclassified (green) samples. **A**. Performance of CanID on ST. SJCloud TestData1: overall accuracy = 0.989 (*n* = 181), with 6 samples below the threshold. **B**. Performance of CanID on ST TARGET TestData2. TestData2 comprised all external TARGET samples. Overall accuracy was 0.989 (*n* = 451), with no samples below the threshold. NBL achieved perfect accuracy (168/168). OS showed 98.9% accuracy (87/88 correctly classified, 1 misclassified), RT showed 98.5% accuracy (64/65 correct, 1 missed), and WT showed 97.7% accuracy (127/130 correctly classified, 3 misclassified). **C**. Performance of CanID on ST clinical pilot TestData3. All predictions above the scoring threshold were correct (*n* = 20), with 1 sample below the threshold. The bar chart shows the distribution of filtered samples (blue), correctly classified (orange), and misclassified samples (green). ACC, adrenocortical carcinoma; ARMS, alveolar rhabdomyosarcoma; ERMS, embryonal rhabdomyosarcoma; EWS, ewing sarcoma; HB, hepatoblastoma; MEL, melanoma; NBL, neuroblastoma; OS, osteosarcoma; RBL, retinoblastoma; RT, rhabdoid tumor; SYNS, synovial sarcoma; THPA, thyroid papillary carcinoma; WT, wilms tumor; HM, hematologic malignancy.

To evaluate CanID’s performance, we built a pediatric ST classifier using a reference set of 456 samples from the ST dataset (450 from SJCloud and 6 RT samples from ZERO). We evaluated the classifier in the validation group [ST samples in TestData1 (*n* =181), with 2 RT samples from ZERO] and achieved an accuracy of 0.989]. CanID correctly predicted the subgroups for 173 test samples with confidence scores above the threshold (175 samples), while 6 samples fell below the threshold (**Figure 2**A; Tables S3 and S15).

We evaluated the performance of the ST classifier in the NCI TARGET cohort (TestData2), an external pediatric pan-cancer dataset. CanID achieved an accuracy of 0.989 (*n* = 451) and successfully predicted all samples above the confidence threshold (Figure 2B; Tables S3 and S16). We then evaluated the performance using the ClinGen Pilot cohort (TestData3), an independent pan-cancer cohort with detailed clinical and molecular characterizations. The TestData3 contained 20 ST samples, and CanID correctly predicted the subtypes of 19 samples, whereas the remaining sample fell below the confidence threshold (Figure 2C; Table S3). When we compared CanID with OTTER, a recently published RNA-seq based classifier for pediatric cancer [19], CanID outperformed OTTER in terms of both sensitivity and selectivity, producing a higher number of correct predictions (19 in CanID *vs*. 7 in OTTER) and a much lower number of incorrect predictions (0 in CanID *vs*. 8 in OTTER; Table S17). Here, selectivity refers to the proportion of predictions that were correct, reflecting the model’s ability to limit classifications to high-confidence cases.

**Figure 3:**
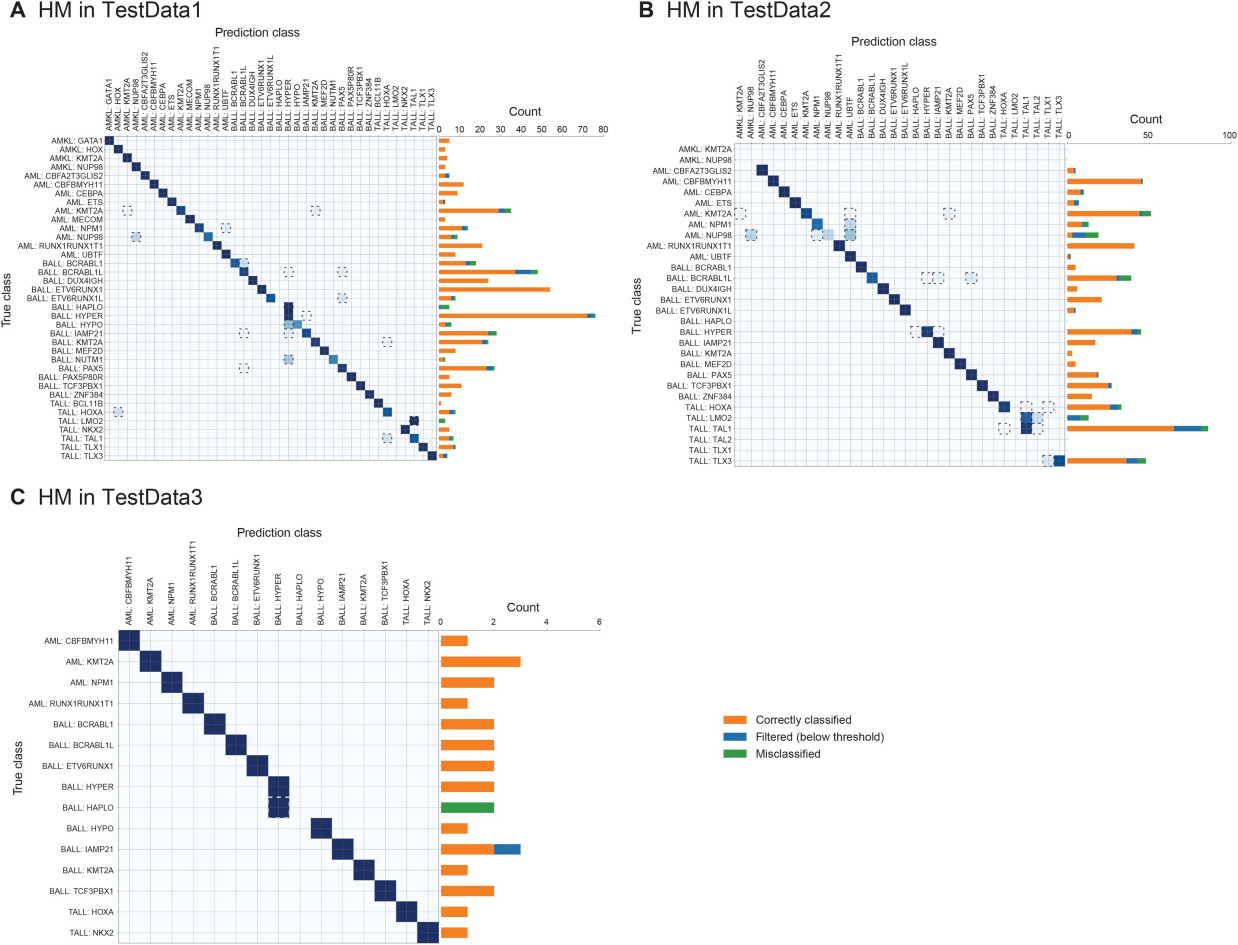
Performance of CanID on HM test sets **A**. TestData1 consisted of 30% reserved SJCloud samples. Performance of CanID on HM; SJCloud TestData1: overall accuracy = 0.936 (*n* = 522), with 38 samples below the threshold. **B**. TestData2 comprised all external TARGET samples. Performance of CanID on HM TARGET TestData2: overall accuracy = 0.920 (*n* = 581), with 66 samples below the threshold. **C**. The TestData3 consisted of clinical pilot samples. Performance of CanID on HM clinical pilot TestData3: overall accuracy = 0.920 (*n* = 28), with 3 samples below the threshold. The bar chart shows the distribution of filtered samples (blue), correctly classified (orange), and misclassified samples (green).

We trained an HM classifier with 1313 samples. Unlike the ST dataset, where tumors were derived from 13 major cancer types with largely distinct tissues, the HM dataset contained 38 related tumor subtypes from three major types (AML with 14 subtypes, B-ALL with 16 subtypes, and T-ALL with 8 subtypes). The inter-type correlation (mean 0.865 +/− 0.041) was much closer to the intra-type correlation (mean 0.910 +/− 0.035) in HM than in ST (inter and intra-type correlations were 0.788 +/− 0.062 and 0.876 +/− 0.061, respectively). The high expression similarity among HM subtypes increased the difficulty of classification (Figure S5), reflected in a larger number of PCA features used in the classifier. When we tested the classifier in TestData1, it achieved an accuracy of 0.936 (*n* = 523 with 485 predicted and 38 below the threshold; **Figure 3**A; Table S15). In TestData2 (TARGET), the classifier achieved an accuracy of 0.920 (*n* = 581 with 515 predicted and 66 below the threshold; Figure 3B; Table S16).

Similarly, CanID achieved an accuracy of 0.920 (*n* = 28 with 25 predicted and 3 below threshold; Figure 3C) in the HM Clinical Pilot cohort (TestData3). It also outperformed OTTER in terms of both sensitivity and selectivity, producing more correct subtype predictions (23 in CanID *vs*. 3 in OTTER) and fewer incorrect subtype predictions (2 in CanID *vs*. 15 in OTTER; Table S17).

### Subtype prediction for samples labeled as not otherwise specified

A subset of samples from the SJCloud and TARGET cohorts (52 out of 1399 in ST and 779 out of 3359 in HM) were initially labeled as not otherwise specified (NOS). To evaluate whether CanID could classify these cases, we analyzed CanID-predicted subtype labels for approximately 70% of samples with confidence scores higher than the subtype-specific thresholds (36 in ST and 555 in HM samples). *PAX5* is a critical regulator of B-cell development, and disrupting its gene function drives B-ALL leukemogenesis [43]. We derived a list of candidate PAX5-subtype specific marker genes by combining the top overexpressed genes (*VIPR2, ERBB2, NELL1*, and *TPBG*) in the PAX5 subtype (*vs*. other B-ALLs) in the training set with those reported in the literature (*PAX5, PRDM15, DENND6B*, and *TOR4A*) [44,45] that were also significantly overexpressed in the training set. CanID predicted B-ALL PAX5 samples in both TestData1 and the NOS group and found that they shared the same transcriptome signatures as those in the training set (Figure S6).

We next evaluated the RMS and NOS subtypes. The two major histological subtypes of RMS, ARMS and ERMS, have significantly different prognoses [46]. Traditionally, pathologists label RMS cases that cannot be further subtyped through histology as NOS. We analyzed 36 such samples from the SJCloud cohort and compared the predictions from CanID and OTTER. Using the ST classifier, CanID predicted 31 samples with a confidence score higher than the threshold (24 ERMS and 7 ARMS) and scored 5 samples below the confidence threshold. OTTER generated predictions for 33 of the 36 samples: 12 were classified as ERMS, 6 as ERMS-like, 6 as ARMS, and 9 as tumor types outside the RMS category. An expert pathologist reviewed these 36 NOS samples and identified sufficient evidence to assign subtypes for an additional 12 samples (10 ERMS and 2 ARMS). Of those, the predictions made by our classifier perfectly matched the expert pathologist’s assignments, while OTTER matched six samples, partially matched three, misclassified two, and made no prediction for one sample (Table S12).

The ability of our classifier to accurately subtype RMS and NOS samples was supported by signature gene expression and fusion gene analysis. *MYOG*, *TFAP2B*, *NOS1*, and *HMGA2* are well-established surrogate markers for fusion status in RMS [47]. *MYOG*, *TFAP2B*, and *NOS1* showed higher expression in the ARMS group, whereas *HMGA2* showed higher expression in the ERMS group. In both the training and testing groups, the expression patterns for *MYOG, TFAP2B,* and *NOS1* matched the ARMS-specific markers reported in the literature. Likewise, the expression pattern for *HMGA2* in the training and testing groups matched expectations, with *HMGA2* upregulated in the ERMS group compared to the ARMS group. This pattern also appeared in the predicted subtype within the RMS, NOS group, with predicted ARMS samples showing upregulation of *MYOG*, *TFAP2B,* and *NOS1*. Similarly, *HMGA2* expression was also elevated in RMS and NOS samples predicted to be ERMS (Figure S7).

Recent research suggests that recurrent *PAX3/7–FOXO1* fusions serve as the primary drivers of ARMS, and most fusion-negative ARMS are actually ERMS [48]. Therefore, we used Fuzzion2 [40], an in-house algorithm for detecting known fusions at high sensitivity in RNA-seq data (see details in Method) on this RMS and the NOS group. Notably, *PAX3/7–FOXO1* fusions appeared in all samples classified as ARMS (7 samples), but in none of the samples classified as ERMS (24 samples). In contrast, OTTER classified subtypes for 33 samples in this group, designating 18 as RMS and matching the fusion status (12 ERMS and 6 ARMS), producing 6 partially matched predictions (all ERMS) and 9 incorrect predictions (8 ERMS and 1 ARMS; Table S12).

Interestingly, the *PAX3/7–FOXO1* fusion was also detected in SJRHB010463_M1, an ARMS tumor with low purity (< 20%) [49] and one of the three unclassified samples from CanID. This finding highlights the potential for further improving the prediction performance by incorporating additional RNA-seq analyses, such as fusion detection.

### Multi-omics profile of a RT classified as NBL with a high confidence score

**Figure 4:**
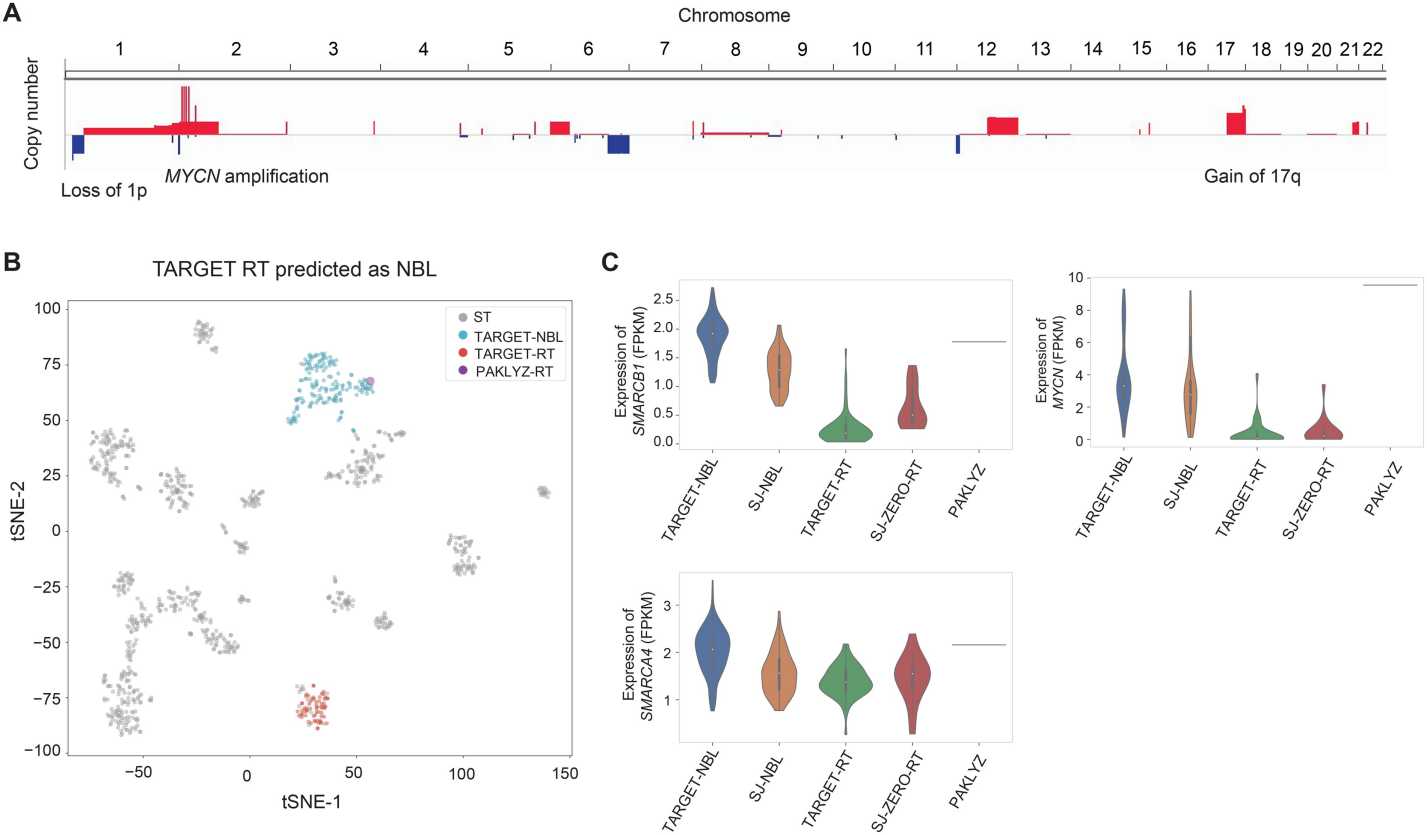
Evidence that TARGET RT sample PAKLYZ is mislabeled **A**. Copy-number profile of TARGET sample PAKLYZ showing three signature NBL CNVs; (1) gain of 17q, (2) loss of 1p, and (3) MYCN amplification on chromosome 2 (> 50 copies) accompanied by MYCN overexpression. **B**. tSNE projection demonstrating that PAKLYZ clusters with NBL samples rather than RT samples. **C**. Expression analysis of the outlier sample showing elevated *SMARCB1* and *SMARCA4* expression along with high *MYCN* expression. CanID classified this sample as NBL with high confidence (score = 0.969). CNV, copy-number variant; tSNE, t-distributed stochastic neighbor embedding.

The TARGET sample PAKLYZ was labeled as RT, but CanID predicted it as NBL with high confidence (0.969). This incorrect prediction had the highest CanID confidence score among all other incorrectly predicted ST samples. To investigate this potential misclassification, we analyzed the whole-genome sequencing (WGS) data and the expression profile of PAKLYZ in depth. RTs are driven by bi-allelic loss of the switch/sucrose non-fermentable (SWI/SNF) complex involving *SMARCB1* or *SMARCA4* [50,51]. However, the WGS analysis of PAKLYZ from published literature [11] did not detect any mutation or copy-number loss of *SMARCB1* or *SMARCA4*. This finding aligned with a previous report that assigned PAKLYZ as a *SMARCB1*-intact sample based on its elevated *SMARCB1* and *SMARC4* expression, as well as clustering with normal control samples instead of RT samples using H3K27me3 signatures [52]. Importantly, our WGS analysis identified three copy number variants (CNVs) typically found in NBL: (1) gain of 17q, (2) loss of 1p, and (3) a *MYCN* amplicon, a well-established key driver of NBL tumorigenesis [53], with an estimated > 50 copies accompanied by *MYCN* overexpression (**Figure 4**A). We observed that PAKLYZ clustered with NBL samples rather than RT samples on a tSNE projection (Figure 4B). Finally, expression analysis of PAKLYZ showed elevated *SMARCB1* and *SMARCA4* expression along with high *MYCN* expression (Figure 4C). Therefore, evidence from the mutational landscape, the expression profile, and a prior published epigenetic signature all support CanID’s classification of this case as NBL, suggesting that the initial label was likely incorrect.

### Robustness against noise in the training samples

In the current study, the RT sample PAKLYZ from the TARGET cohort may represent an example of mislabeling in OMICs data, which can arise from human errors throughout the experimental process, including sample collection, transportation, data generation, and analysis. Researchers have found that mislabeling is relatively common in published expression datasets. Using a few sex-specific markers, two studies independently identified gender-mismatched samples in 40%–46% of the transcriptomic datasets, although the overall percentage of mismatched samples was relatively low [54]. In supervised learning schemes, mislabeled samples (or label noise) in the training data often deteriorate classifier performance [55]. Researchers have proposed approaches to identify and “correct” labels for potentially mislabeled samples [56,57]. However, without a thorough pathology review of these suspected cases, such attempts are less appropriate in tumor classification schemes. Therefore, we tested the robustness of CanID against mislabeled samples. We permuted the labels in 5%, 10%, 15%, 20%, 30%, 40%, and 50% of the training samples and built a CanID model for each permuted training set. For each error level, we repeated the process 10 times to evaluate performance.

**Figure 5:**
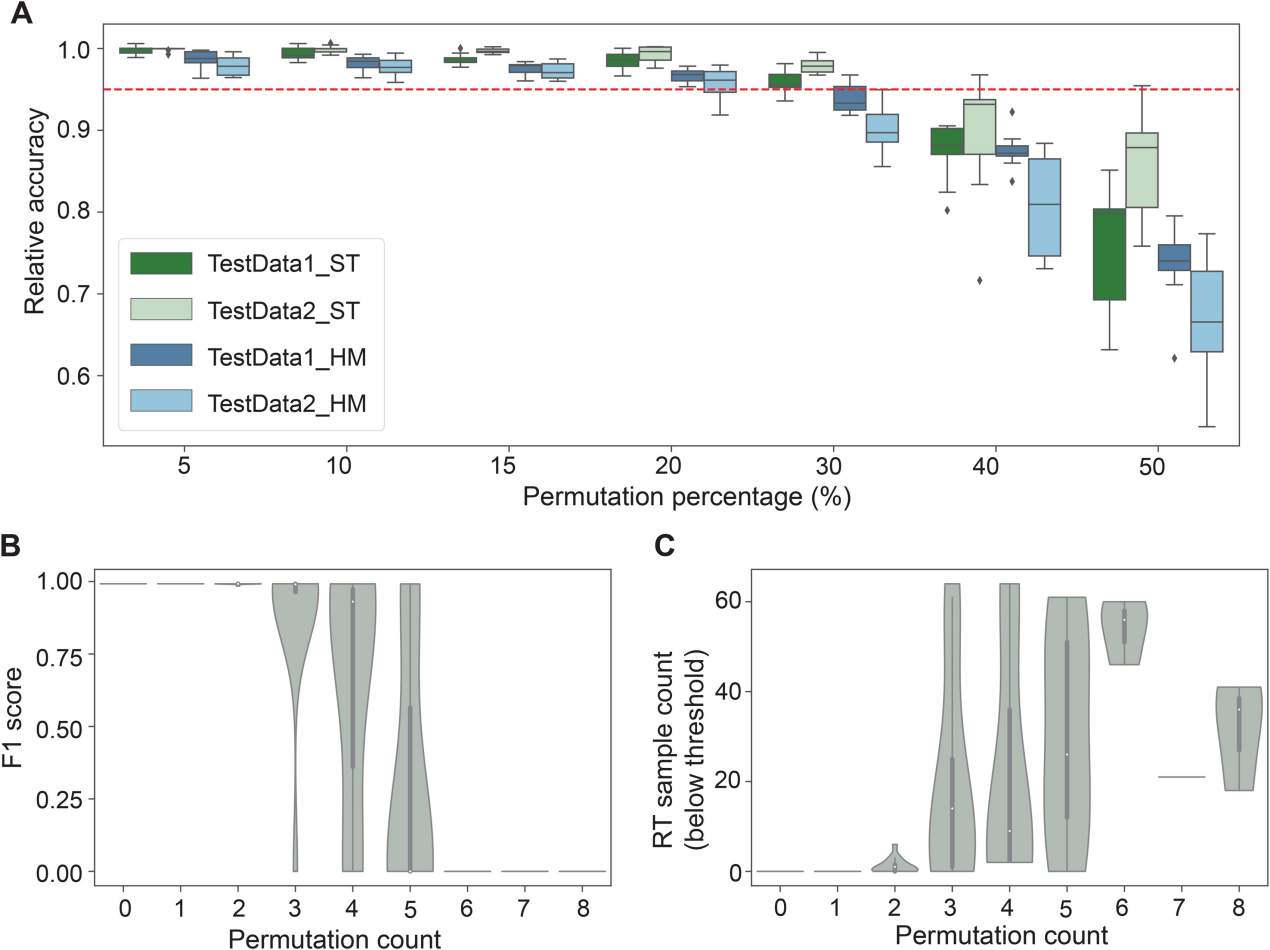
Robustness analysis of CanID **A**. Overall performance of ST and HM CanID models on TestData1 and TestData2 under varying levels of sample label permutation, normalized to the original accuracy. The red dashed line indicates a relative accuracy of 95%. **B**. Effect of RT-specific label noise on the F1 score. **C**. Effect of RT-specific label noise on the number of RT samples below the threshold.

Although the overall accuracy of the CanID ST model decreased with an increase in label noise, it remained comparable to the original classifier (within 5% of the original accuracy; **Figure 5**A), with approximately 10% of the predicted sample labels falling below the scoring threshold (Figure S8). We observed this behavior in two large test sets with up to 30% permuted training samples (we excluded TestData3 due to its small sample size). The performance deteriorated substantially (lower accuracy and higher proportion below threshold) when the error level exceeded 30%. Similarly, in the more challenging HM dataset, the classification accuracy remained relatively stable (within 5% of the original accuracy) with up to 20% label noise but declined sharply when the noise exceeded 20% (Figure 5A, Figure S8).

Real-world classification scenarios often involve both noisy labels (mislabeled training samples) and class imbalance. Mislabels in the minor classes are more detrimental because minor classes are often under-learned, and the decision boundary is more susceptible to mislabeled samples [58]. In this study, the RT class in the ST CanID model had 11 samples in the training set and 65 samples in the independently collected TARGET cohort (TestData2), providing a clear example to examine the model’s behavior in this challenging setting. We analyzed the effects on RT classification from both the overall level of noise and RT-specific noise (the number of mislabeled RT samples) for individual runs. The F_1_ score for the RT classification in the TARGET cohort declined strongly and significantly with increasing RT-specific noise (β = −0.16, *P =* 5.02 × 10^−15^, linear regression), but was not affected by the overall noise level (dropped during the stepwise model selection procedure). In general, the CanID model tolerated approximately 30% RT-specific label noise without a substantial loss in performance, which is comparable to the tolerance to the overall performance (Figure 5B). Similarly, RT-specific noise, but not the overall error rate, contributed significantly and positively (β = 6.53, *P =* 1.60 × 10^−8^, linear regression) to the number of RT samples below the threshold, because the confidence score dropped below the run-specific threshold (Figure 5C).

### Robustness to reference build and alignment

We evaluated the robustness of classifier performance across differences in genome build, annotation version, and aligner using the human genome versions 38 (hg38) and 19 (hg19). We trained CanID with reads mapped to hg38 using the STAR aligner in two-pass mode and quantified expression with HTSeq counts generated from GENCODE v31. To assess performance under alternative alignment strategies, we identified 25 samples with paired alignments — Burrows–Wheeler Aligner (BWA) to hg19 (GENCODE v19) and STAR to hg38 (GENCODE v31) — enabling within-sample comparison across reference build, annotation release, and aligner (see Method). The dataset contained one ST case [adrenocortical carcinoma (ACC)] and multiple HM subtypes, including AML (CBFA2T3-GLIS2, NUP98, GATA1, KMT2A, and HOX), and B-ALL subgroups (MEF2D, PAX5P80R, and ZNF384) (Table S21). All processing methods achieved an accuracy of 1.0, with only five samples per method filtered (Table S22). These findings indicate that classifier accuracy was unaffected by changes in genome build, annotation version, or aligner.

### Exploratory analysis of prediction for samples from rare subtypes not included in the training

A common assumption for classifiers or supervised learning schemes is that the set of classes (or labels) encountered in deployment matches the set included in training (*i.e.*, closed-set assumption). However, in real-world applications, most classifiers encounter classes or labels not covered in the training (*i.e.*, open-set conditions). Although specialized algorithms are preferred to address this more realistic but challenging scenario [59–61], we evaluated the performance of our ST classifier, which offers stronger inter-class separations, using samples from rare cancer subtypes.

We analyzed 202 samples in the SJCloud Rare ST cohort, covering 97 different subtypes (1–8 samples per subtype). Unlike the minimal filtering in TestData1 (3%, 6 out of 181 below threshold), CanID did not classify half of the samples from rare subtypes (52%, 105 out of 202). Among the predicted samples, we observed several patterns in which most samples from a specific subtype were predicted as a trained subtype: (1) rare subtypes that were subclasses of an existing trained subtype (*e.g.*, ganglioneuroblastoma predicted as neuroblastoma, botryoid-type embryonal rhabdomyosarcoma and spindle cell rhabdomyosarcoma predicted as ERMS [46], chondroblastic osteosarcoma and osteoblastic osteosarcoma predicted as osteosarcoma, *etc.*); (2) rare subtypes that potentially shared a similar cell of origin with an existing trained subtype (*e.g.*, adrenal cortical adenoma predicted as adrenal cortical carcinoma, hepatocellular carcinoma and hepatic focal nodular hyperplasia predicted as hepatoblastoma, aneurysmal bone cyst predicted as osteosarcoma, pheochromocytoma predicted as neuroblastoma, follicular thyroid cancer predicted as papillary thyroid cancer, *etc.*); and (3) subtypes that shared similarity with an existing trained subtype (*e.g.*, ectomesenchymoma predicted as ERMS [62], clear cell sarcoma predicted as melanoma [63], *etc.*).

We observed a similar pattern in the TARGET ST cohort, where 92% (12 out of 13) of clear cell sarcoma of the kidney samples fell below the threshold, with the remaining sample predicted as Wilms tumor — potentially indicating a shared cell of origin. Likewise, in the Clinical Pilot ST cohort, 86% (6 out of 7) of samples were below the CanID threshold.

Despite CanID’s overall accuracy in pediatric STs, two SJ-Cloud samples from TestData1 and five TARGET ST samples were misclassified with confidence scores exceeding the predetermined threshold. These included the previously discussed PAKLYZ sample (Figure S9; Table S16). Specifically, one ERMS sample (SJRHB031519_D1) was misclassified as ARMS; one ACC sample (SJACT030437_D1) was misclassified as Wilms tumor (WT); three WT samples (CAAAAM, PAJMVC, and PAJNVX) were misclassified as ERMS; and one OS sample (PASEFS) was misclassified as ERMS. To further understand why multiple WT samples were misclassified as ERMS, we identified PCA features with strong significant differences between ERMS and WT in the training samples — PC3 and PC4 (Table S9). Among these differentiating PC features, the three misclassified WT TARGET samples clustered more closely with the distribution of ERMS than with that of the WT subtype. Figure S10 depicts a violin plot of PC3 and PC4 for WT and ERMS samples, indicating that the misclassified WT cases align with ERMS samples. Figure S11 depicts a scatter plot of PC3 *vs.* PC4 showing the misclassified WT cases align with ERMS samples. A GSEA leading-edge analysis of the gene weights on PC3 revealed strong enrichment of myogenic differentiation-related pathways — a hallmark of RMS tumors — in the negative leading edge (Figure S12; Table S23). Notably, the three misclassified samples were clear outliers showing negative PC3 values. Figure S13 is a violin plot of PC3 split across training and testing ERMS and WT samples, which showed the missed WT samples (CAAAAM, PAJMVC, and PAJNVX) have negative PC3 values (−32.7 ± 13.1), aligning with ERMS training samples (−24.0 ± 26.8) rather than WT samples (38.6 ± 24.6). In contrast, most other WTs exhibited strong positive PC3 values (Figures S11 and S13). Moreover, we observed elevated expression of *MYOD1* and *MYOG*, master transcription regulators of myogenic differentiation in these samples (Figure S14). These observations suggest that these tumors may represent post-therapy (chemotherapy and/or radiotherapy) tumors with induced myogenic differentiation [64].

## Discussion

We developed CanID, a general (sub)type classification scheme for tumors, that uses RNA-seq feature counts as the sole input for classifying tumor subtypes. We accomplished this by establishing preprocessing models that include data normalization, batch correction, and feature reduction to transform the test data. By combining the outputs from five strong classifiers, we constructed an ensemble classification model from the transformed features to predict tumor subtypes and assign an associated confidence score to each prediction. CanID achieved high accuracy in testing samples from the same source (split into separate training and test sets) and independent datasets. Importantly, in a comparison with a state-of-the-art prediction algorithm (OTTER) using a well-characterized testing set (ClinGen Pilot) and the SJCloud RMS NOS set, CanID produced better predictions for subtypes included in the training and correctly identified half of the tumors from rare subtypes not included in the initial training, as shown below the threshold.

Incorrect sample annotations frequently occur in transcriptomic data and pose a potential threat to classification schemes. We evaluated the performance of CanID under various levels of intentionally introduced label noise (5%–50%). We demonstrated that CanID models maintain robust performance with up to 30% label noise in the ST classifier and 20% in the HM classifier — exceeding the reported overall error rates observed in previous transcriptomic studies (4.1% including misclassified and unconfident samples [65], and 2.04% in cancer datasets [54]). Importantly, our analysis in RT further demonstrated that the performance of minor classes in the training phase showed a similar error tolerance.

Model robustness remains a major challenge for advanced machine learning algorithms, including deep learning. Studies have demonstrated that minor perturbations to the input of a machine learning model can have devastating effects on its predictions [66]. In tumor classification schemes, transcriptome-based classifiers may encounter challenges due to subtle differences between the two major RNA-seq platforms: the mRNA-seq protocol, which enriches mRNA with poly(A) tails, and the total stranded RNA protocol, which depletes rRNAs. Despite research showing high concordance across these different RNA-seq library protocols [67], a recent study excluded all samples prepared with a total stranded library protocol because of apparent and consistent batch effects across tumor types [19]. However, to improve the applicability of the classifier, we aim to maintain robust performance across different library preparation protocols. By employing a feature extraction scheme that focuses on the global transcriptomic pattern, CanID maintains strong performance in subtype prediction, not limited to test cases with comparable mRNA-seq/total RNA-seq composition as the training data. The TARGET test data, generated solely by mRNA-seq, achieved comparable performance (Table S24). Moreover, for subtypes not included in the initial training, CanID correctly labeled most of them as unclassifiable. Finally, the high performance of the classification scheme in NOS rhabdomyosarcoma further demonstrates CanID’s potential to assist pathologists in determining tumor subtypes for challenging samples.

The subtype labels used for training classifiers can originate either from clinical diagnoses or clustering analyses. While the latter approach offers the advantage of revealing potential novel subtypes that are not well characterized in current clinical practice, it also presents conceptual and technical challenges, such as confusion among distinct subtypes with similar transcriptome signatures. For example, the transcriptome profiles of near-haploid BALL samples and hyper-diploid BALL samples are highly similar, potentially due to the relative overrepresentation of the same set of chromosomes [16]. Consequently, unbiased clustering may mistakenly assign them to a single cluster despite distinct clinical risk stratifications between them [68]. In the current study, we followed the WHO classification scheme and supplemented it with expert pathologist review of clinical and molecular testing, with the expectation that our classifiers would deliver clinically relevant performance.

In the current implementation, we explicitly use the minimal input requirement — an array of feature counts for individual genes — to maximize model portability. However, ancillary information computable from the raw sequencing files can further aid in classifying tumor subtypes in challenging samples, as demonstrated by the detection of a PAX3::FOXO1 fusion in SJRHB010463_M1, a fusion-positive ARMS sample with low tumor purity [49]. In contrast, barcode hopping remains an in-gene fusion-based classification scheme, in which multiple subtype-defining oncogenic fusions occur in a small fraction of patient samples. For example, four samples included in this study contained multiple fusions (SJBALL020141 and SJBALL020142 with MEF2D::DAZAP1 [majority] and KMT2A::MATR3 [minority], SJCBF124 and SJCBF149 with RUNX1::RUNX1T2 [majority] and CBFB::MYH11 [minority]) [69]. Since CanID relies on the entire transcriptome rather than a few “drivers”, it correctly predicted, with high confidence, all four samples as belonging to the class defined by the majority fusion. Therefore, both whole transcriptome-based (such as CanID) and fusion-based classification schemes can strengthen classification power in challenging cases for the other scheme.

To integrate CanID with additional data modalities, such as mutation profiles, expression signatures, and fusion status, these inputs must first be transformed into model-ready features and combined with the PCA features derived from CanID. By concatenating these categorical and continuous variables with the PCA features, the classifier can leverage both broad transcriptomic variation and targeted molecular markers to refine subtype discrimination during training. This approach has the potential to improve classification accuracy and the resolution of discrete subtypes.

The model’s consistent misclassification of the T-ALL LMO2 subgroup reflects the underlying biological similarity between T-ALL TAL1 and T-ALL LMO2 leukemias. Euclidean distance analysis of training sample expression profiles shows that T-ALL TAL1 samples more closely resemble T-ALL LMO2 samples than other T-ALL subgroups, indicating highly similar transcriptional programs. The tSNE projection further supports this relationship, with T-ALL LMO2 samples clustering alongside T-ALL TAL1 cases rather than forming a distinct group (Figure S15). This clustering suggests that their global expression profiles are nearly indistinguishable in the reduced-dimensionality space used by the model. Biologically, the *TAL1* and *LMO2* genes encode proteins that form a multiprotein complex with other hematopoietic transcription factors to drive T-ALL leukemogenesis [70]. Studies have shown that *LMO2* is required for proper *TAL1* target binding, and the two factors cooperate to enforce a shared oncogenic program in both patient samples and mouse models [71–73]. Because these two subtypes are transcriptionally overlapping and occupy the same region in the tSNE space, the model lacks the power to distinguish T-ALL LMO2 from T-ALL TAL1 tumor types.

A limitation of the current implementation of CanID is the requirement of at least 10 samples per subtype for model construction. Consequently, rare tumor subtypes, such as clear cell sarcoma of the kidney (CCSK), while clinically relevant, cannot be integrated owing to an insufficient number of samples. We are actively expanding the number of cases for rare subtypes to broaden the classification scope of pediatric STs. We plan to regularly update our CanID models as additional data becomes available. Furthermore, users can easily train their own models using the open-source code and instructions available on GitHub (https://github.com/chenlab-sj/CanID).

An important potential application of this work is the reclassification of legacy transcriptomic datasets. Many older studies relied solely on histopathology or limited molecular markers, resulting in incomplete or outdated subtype assignments. Applying CanID to these archived RNA-seq datasets would enable subtype prediction based on transcriptomic patterns captured through PCA-derived features. This approach could rapidly detect potential misclassifications, harmonize subtype definitions across cohorts, and enhance the accuracy of meta-analyses. In turn, it would facilitate cross-study comparisons and unlock the potential of underutilized archival datasets for precision oncology research.

## Conclusion

We developed a general classification scheme capable of generating classifiers for specific purposes. Applications across three pediatric cancer datasets with clinically relevant cancer type definitions demonstrated its robustness to sequencing library preparation protocols and batch effects, portability, and accuracy based on biologically interpretable features. The trained classifier distinguished most previously unclassifiable samples — 36 of 52 in ST and 555 of 779 in HM. With clear biological interpretability and high prediction accuracy, our pan-cancer transcriptome-based classification scheme offers a valuable tool to support tumor diagnosis and enable clinically meaningful stratification of tumor types in pediatric patients with malignancies.

## Code availability

CanID is available on GitHub at https://github.com/chenlab-sj/CanID, along with documentation.

## CRediT author statement

**Daniel K. Putnam:** Data curation, Formal analysis, Investigation, Methodology, Resources, Software, Validation, Visualization, Writing – original draft, Writing – review & editing. **Alexander M. Gout:** Data curation, Formal analysis, Investigation, Resources, Visualization, Writing – original draft, Writing – review & editing. **Delaram Rahbarinia:** Data curation, Investigation, Resources, Writing – review & editing. **Meiling Jin:** Formal analysis, Investigation, Resources, Writing – review & editing. **David Finkelstein:** Formal analysis, Resources, Writing – review & editing. **Xiaotu Ma:** Formal analysis, Resources, Writing – review & editing. **Jinghui Zhang:** Conceptualization, Formal analysis, Project administration, Supervision, Writing – original draft, Writing – review & editing. **David A. Wheeler:** Conceptualization, Formal analysis, Project administration, Supervision, Writing – original draft, Writing – review & editing. **Larissa V. Furtado:** Conceptualization, Formal analysis, Project administration, Writing – original draft, Writing – review & editing. **Xiang Chen:** Conceptualization, Data curation, Formal analysis, Funding acquisition, Methodology, Project administration, Supervision, Writing – original draft, Writing – review & editing. All authors have read and approved the final manuscript.

## Competing interests

There are no conflicts of interest.

## Supplementary Material

qzaf122_Supplementary_Data
