# Supplementary material for: CanID: A Robust and Accurate RNA-seq Expression-based Diagnostic Classification Scheme for Pediatric Malignancies
Source: Genomics Proteomics Bioinformatics. 2025 Nov 29;23(6):qzaf122. doi: 10.1093/gpbjnl/qzaf122 (PMC13222492; doi:10.1093/gpbjnl/qzaf122)
Supplement: qzaf122_Supplementary_Data [file qzaf122_supplementary_data.zip › Supplementary material captions.docx]

**Supplementary material**

**Figure S1 Pairwise scatter plots of the top nine principal components (PCs) illustrating tumor class separation**

Each point represents an individual tumor training sample, colored according to its class. The figure displays all 36 pairwise combinations of the top nine PCs. These leading PCs, identified through ANOVA-based ranking, exhibit discriminatory power, with distinct clustering patterns observed for tumor classes across multiple component pairings.

**Figure S2 Feature set selection for tumor classification**

**A.** PCA80 was selected as the optimal feature set for ST, non-CNS solid tumors, while (**B**) PCA85 was chosen for HM, hematologic malignancies. In contrast, higher-dimensional feature sets—PCA95 and PCA99—showed a pronounced decrease in performance for HM, with mean accuracies of 0.745 ± 0.008 and 0.555 ± 0.007, respectively**. C.** Comparative results across all HM PCA feature sets are shown.

**Figure S3 Principal component patterns in the solid tumor cohort**

Distinct tumor types are characterized by unique distributions along specific principal components. NBL and RBL exhibit strong positive PC1 values. MEL shows strong negative PC2 values, whereas ERMS, ARMS, and WT show strong positive PC2 values. Along PC3, ERMS and ARMS display strong negative values, while HB, THPA, and WT show strong positive values.

**Figure S4 PC1 developmental and PC2 immune/myogenic signatures**

Using the weighted loadings for Solid-Tumor PC1 and PC2 we performed GSEA to identify biological processes associated with genes driving these components. PC1 was enriched for developmental pathways, reflecting variation in tumor lineage and differentiation. PC2 showed immune-related signatures on the negative leading edge and mitotic and myogenic pathways on the positive leading edge. Panels A and B display enriched gene sets for up-weighted genes, while Panel C shows an enriched set for down-weighted genes.

**Figure S5 Hematologic dataset shows a narrower range of inter- and** **intra-class correlation scores**

The solid tumor cohort exhibits an inter-type correlation of 0.788 ± 0.062 and an intra-type correlation of 0.876 ± 0.061, whereas the hematologic malignancy cohort demonstrates higher correlations, with an inter-type correlation of 0.865 ± 0.041 and an intra-type correlation of 0.910 ± 0.035.

**Figure S6 Expression patterns of candidate PAX5 subtype marker genes**

PAX5, a key regulator of B cell development, is frequently disrupted in B-ALL, B cell acute lymphoblastic leukemia. Candidate PAX5-subtype marker genes were identified by combining the top overexpressed genes in the PAX5 subtype versus other B-ALLs in the training set (*VIPR2*, *ERBB2*, *NELL1*, *TPBG*) with literature-reported genes (*PAX5*, *PRDM15*, *DENND6B*, *TORA*). CanID-predicted subtypes (PAX5 *vs*. B-ALL) in the NOS group mirrored the expression patterns observed in both the training and TestData1 sets.

**Figure S7 Expression pattern of rhabdomyosarcoma surrogate markers for fusion status**

Violin plots of train, test, and NOS, not otherwise specified gene expression for *MYOG*, *TFAP2B*, *NOS1*, and *HMGA2*. *MYOG*, *TFAP2B*, and *NOS1* have higher expression in ARMS, alveolar rhabdomyosarcoma, while *HMGA2* has higher expression in ERMS, embryonal rhabdomyosarcoma. The CanID predicted subtype (ARMS *vs*. ERMS) in the NOS column mirror the expression patterns seen in the train and TestData1 set.

**Figure S8 Performance robustness of solid tumor and hematologic malignancy** **CanID models under increasing sample label permutations**

Overall performance on testset1 and testset2 is shown as the fraction of samples filtered across varying permutation percentages. The fraction filtered increases with higher permutation thresholds. The red dashed line marks a 10% filtering rate.

**Figure S9 Plots of misclassified solid tumor samples cluster with their predicted type**

Testset1: One ACC, adrenocortical carcinoma sample (SJACT030437_D1) was misclassified as WT, Wilms tumor and one ERMS, embryonal rhabdomyosarcoma sample (SJRHB031519_D1) was misclassified as ARMS, alveolar rhabdomyosarcoma. Testset2: Three WT samples (CAAAAM, PAJVMC, PAJNVX) were classified as ERMS, one OS, osteosarcoma sample (PASEFS), was classified as ERMS and one RT, rhabdoid tumor sample (PAKLYZ) was classified as NBL, neuroblastoma. The t-SNE projections were generated from raw counts converted to FPKM for the input gene set (N=17061).

**Figure S10 Misclassified Wilms tumor cases align with embryonal rhabdomyosarcoma profiles**

Violin plots show the distributions of the principal components that distinguish ERMS, embryonal rhabdomyosarcoma (orange) from WT, Wilms tumor (blue) in the training data. Three TARGET, therapeutically applicable research to generate effective treatments samples—CAAAAM, PAJMVC, and PAJNVX—were labeled WT but predicted as ERMS and are shifted toward the ERMS distribution.

**Figure S11 Principal component analysis highlights Wilms tumor cases misclassified as embryonal rhabdomyosarcoma**

Samples shaded red denote WT, Wilms tumor; blue denotes ERMS, embryonal rhabdomyosarcoma. The three misclassified TARGET samples—CAAAAM, PAJMVC, and PAJNVX—labeled WT but predicted as ERMS, are shown as black points and cluster closer to the ERMS group than to WT.

**Figure S12 Principal component 3 captures myogenic differentiation programs**

GSEA, gene set enrichment analysis show enrichment on the negative leading edge for pathways related to myogenic differentiation. This indicates that principal component 3 reflects variation driven by activation of muscle-lineage developmental programs in tumors.

**Figure S13 Principal component 3 distinguishes Wilms tumor from embryonal** **rhabdomyosarcoma**

Violin plots of PC3, principal component values show that the three misclassified TARGET samples (CAAAAM, PAJMVC, PAJNVX) have negative PC3 values (−32.7 ± 13.1), aligning with embryonal rhabdomyosarcoma training samples (−24.0 ± 26.8) rather than Wilms tumor samples (38.6 ± 24.6).

**Figure S14 Elevated myogenic gene expression observed in Wilms tumor cases misclassified as embryonal rhabdomyosarcoma**

Three Wilms tumor samples from the TARGET cohort—CAAAAM, PAJMVC, and PAJNVX—predicted as embryonal rhabdomyosarcoma show elevated *MYOD1* and *MYOG* expression, consistent with activation of myogenic differentiation programs. *MYOD1* and *MYOG* are master transcriptional regulators of myogenesis.

**Figure S15 LMO2 T-cell acute lymphoblastic leukemia cases align with the TAL1** **subtype**

A t-SNE, t stochastic neighborhood embedding projection of training T-ALL samples derived from principal component features (A) shows TALL-LMO2 tumor classes co-cluster with TALL-TAL1 tumor classes rather than forming a distinct group. A heat map of mean pairwise Euclidean distances among training expression profiles (B) indicates TAL1 is closest to LMO2: the mean TAL1–LMO2 distance is lower than the TAL1–TAL1 within-subtype distance and lower than distances to all other T-ALL subtypes.

**Table S1 Summary of the training, testing, and other groups used in CanID development**

**Table S2 Distribution of tumor samples across cohort source**

**Table S3 Classification performance of CanID across independent test datasets**

**Table S4 Prediction outcomes for rare cases across cohorts**

**Table S5 Summary of clinical and research projects contributing RNA-seq data, including sequencing protocol across solid tumor and hematologic malignancy datasets**

**Table S6 ANOVA-based ranking of solid tumor principal components**

**Table S7 Principal component feature counts required to explain variance in solid tumor and hematologic malignancy datasets**

**Table S8 CanID base model parameters of the ensemble classifier**

**Table S9 Significant principal components in training data between mislabeled classes in TARGET solid tumor samples**

**Table S10 CanID out-of-bag prediction results for training samples**

**Table S11 CanID prediction results for ambiguous or not otherwise specified cases from SJ-Cloud and TARGET cohorts**

**Table S12 CanID and OTTER predictions for SJCloud rhabdomyosarcoma not otherwise specified cases, with pathologist review and fusion 2 status**

**Table S13 CanID prediction results for low-confidence cases where pathologist review identified concerns regarding sample quality, limiting reliability of molecular subtyping**

**Table S14 CanID results for rare subtypes, defined as diseases with fewer than 10 cases, across SJ-Cloud and TARGET cohorts**

**Table S15 CanID predictions for TestData1, comprising approximately 30% of harmonized SJ-Cloud patient samples**

**Table S16 CanID prediction results for TestData2, an independent external validation set from TARGET**

**Table S17 CanID prediction results for TestData3, Clinical Pilot samples, with comparison to OTTER predictions**

**Table S18 CanID prediction results for rare Clinical Pilot cases**

**Table S19 Gene set enrichment analysis of the solid tumor gene loadings for principal component 2**

**Table S20 Gene set enrichment analysis of the solid tumor gene loadings for principal component 1**

**Table S21 CanID prediction results, with comparisons across alternative alignment and genome annotation strategies**

**Table S22 CanID prediction results compared across alternative alignment and genome annotation strategies**

**Table S23 Gene set enrichment analysis of the solid tumor gene loadings for principal component 3**

**Table S24 CanID classification accuracy across tumor subtypes represented by both mRNA capture protocols: polyA-derived and total RNA-derived samples**
