# Supplementary material for: CanID: A Robust and Accurate RNA-seq Expression-based Diagnostic Classification Scheme for Pediatric Malignancies
Source: Genomics Proteomics Bioinformatics. 2025 Nov 29;23(6):qzaf122. doi: 10.1093/gpbjnl/qzaf122 (PMC13222492; doi:10.1093/gpbjnl/qzaf122)
Supplement: qzaf122_Supplementary_Data [file qzaf122_supplementary_data.zip › Supplementary_Figures.pdf]

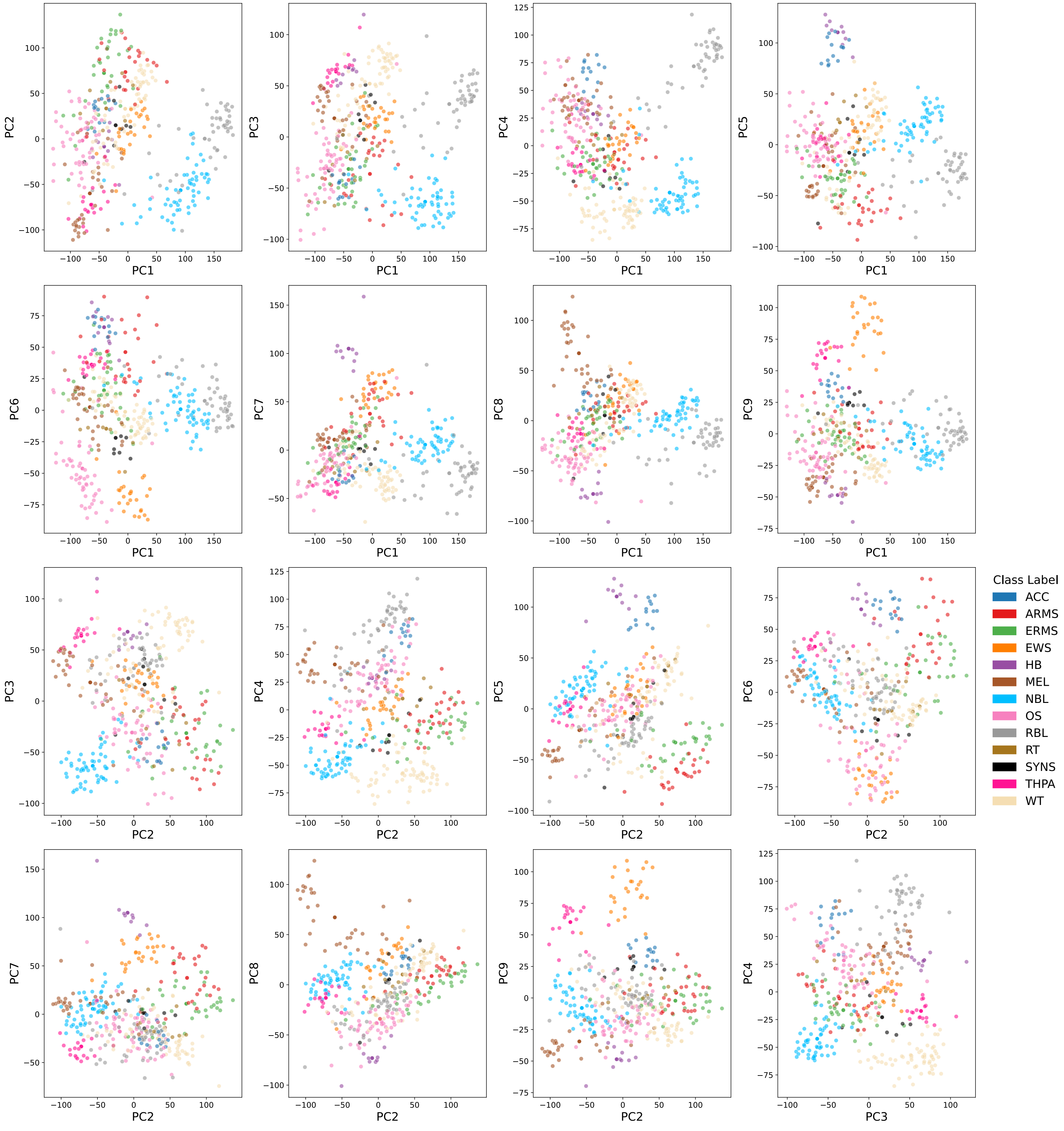

Figure S1 Pairwise scatter plots of the top nine principal components (PCs) illustrating tumor class separation

Each point represents an individual tumor training sample, colored according to its class. The figure displays all 36 pairwise combinations of the top nine PCs. These leading PCs, identified through ANOVA-based ranking, exhibit discriminatory power, with distinct clustering patterns observed for tumor classes across multiple component pairings.

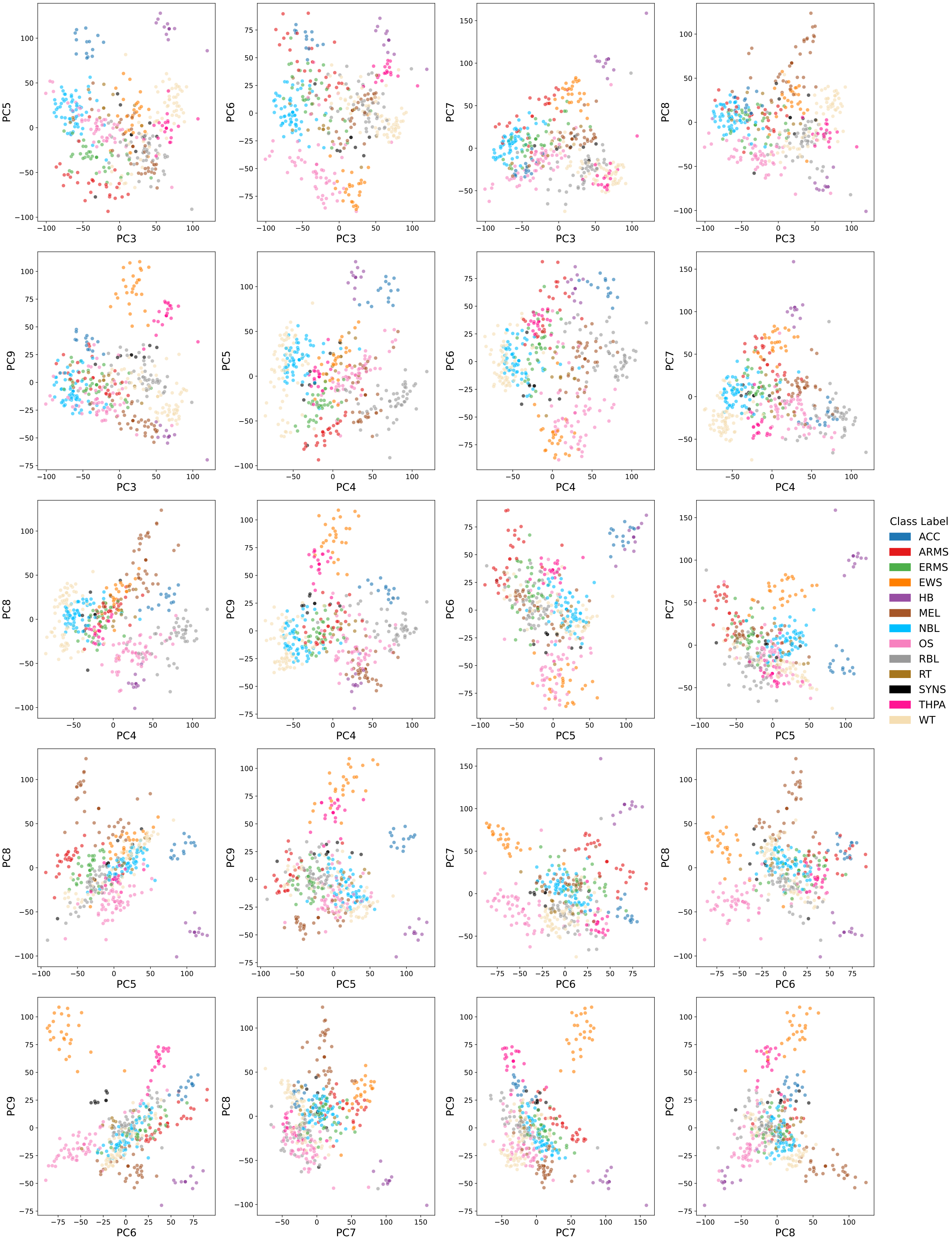

Figure S1 (continued).

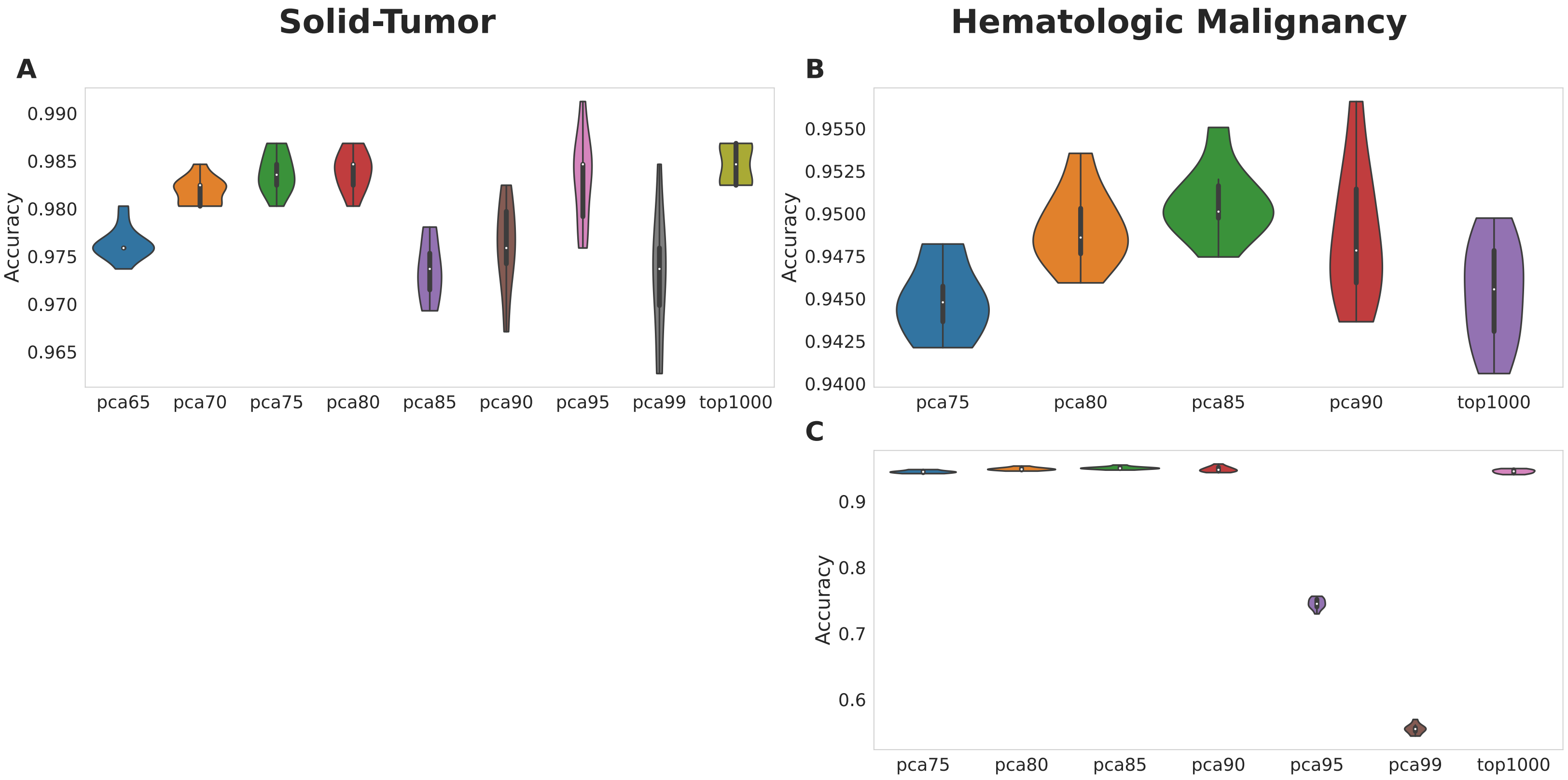

Figure S2 Feature set selection for tumor classification.
A. PCA80 was selected as the optimal feature set for ST, non-CNS solid tumors, while **(B)** PCA85 was chosen for HM, hematologic malignancies. In contrast, higher-dimensional feature sets—PCA95 and PCA99—showed a pronounced decrease in performance for HM, with mean accuracies of 0.745 ± 0.008 and 0.555 ± 0.007, respectively. **C.** Comparative results across all HM PCA feature sets are shown.

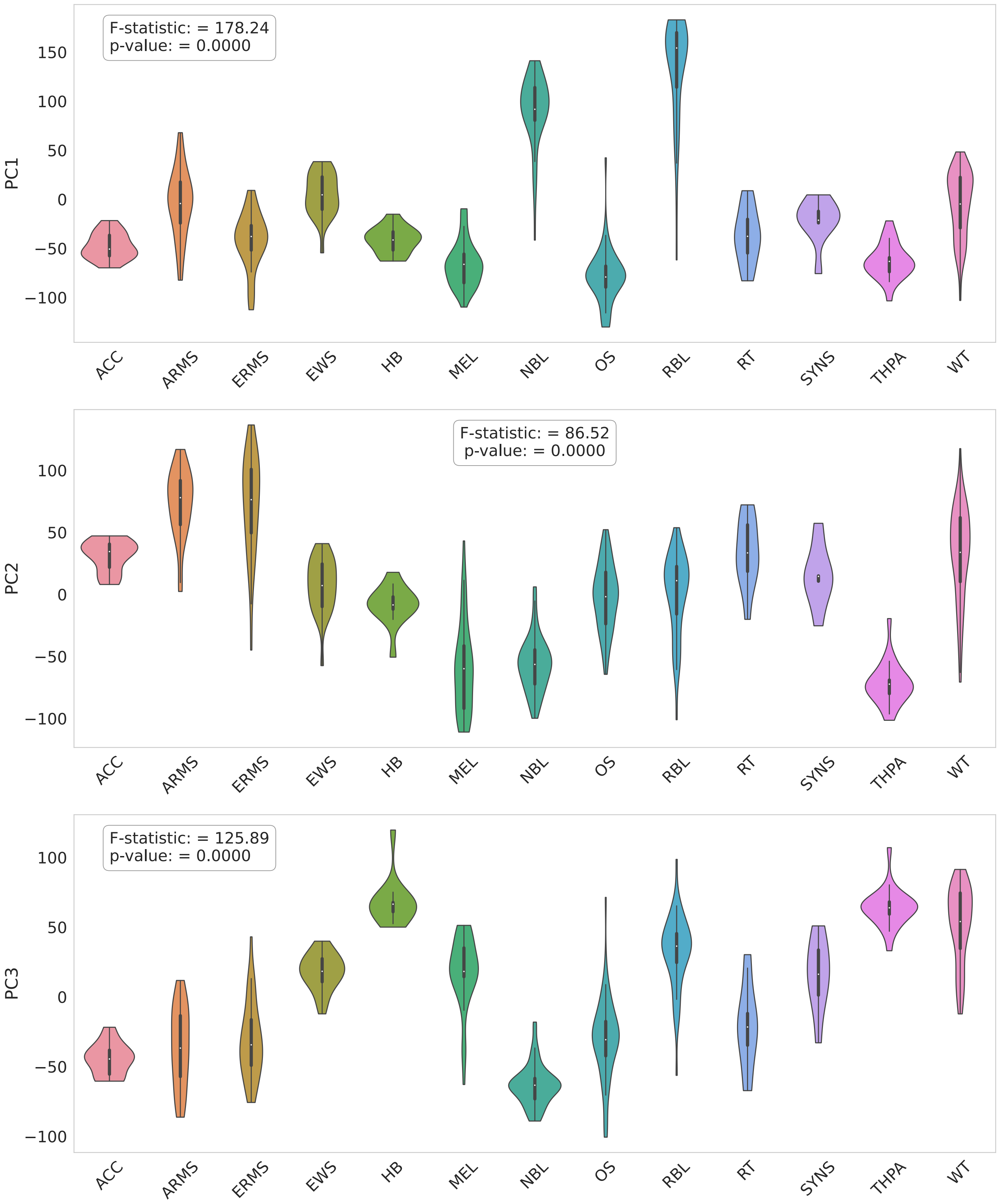

Figure S3 Principal component patterns in the solid tumor cohort
Distinct tumor types are characterized by unique distributions along specific principal components. NBL and RBL exhibit strong positive PC1 values. MEL shows strong negative PC2 values, whereas ERMS, ARMS, and WT show strong positive PC2 values. Along PC3, ERMS and ARMS display strong negative values, while HB, THPA, and WT show strong positive values.

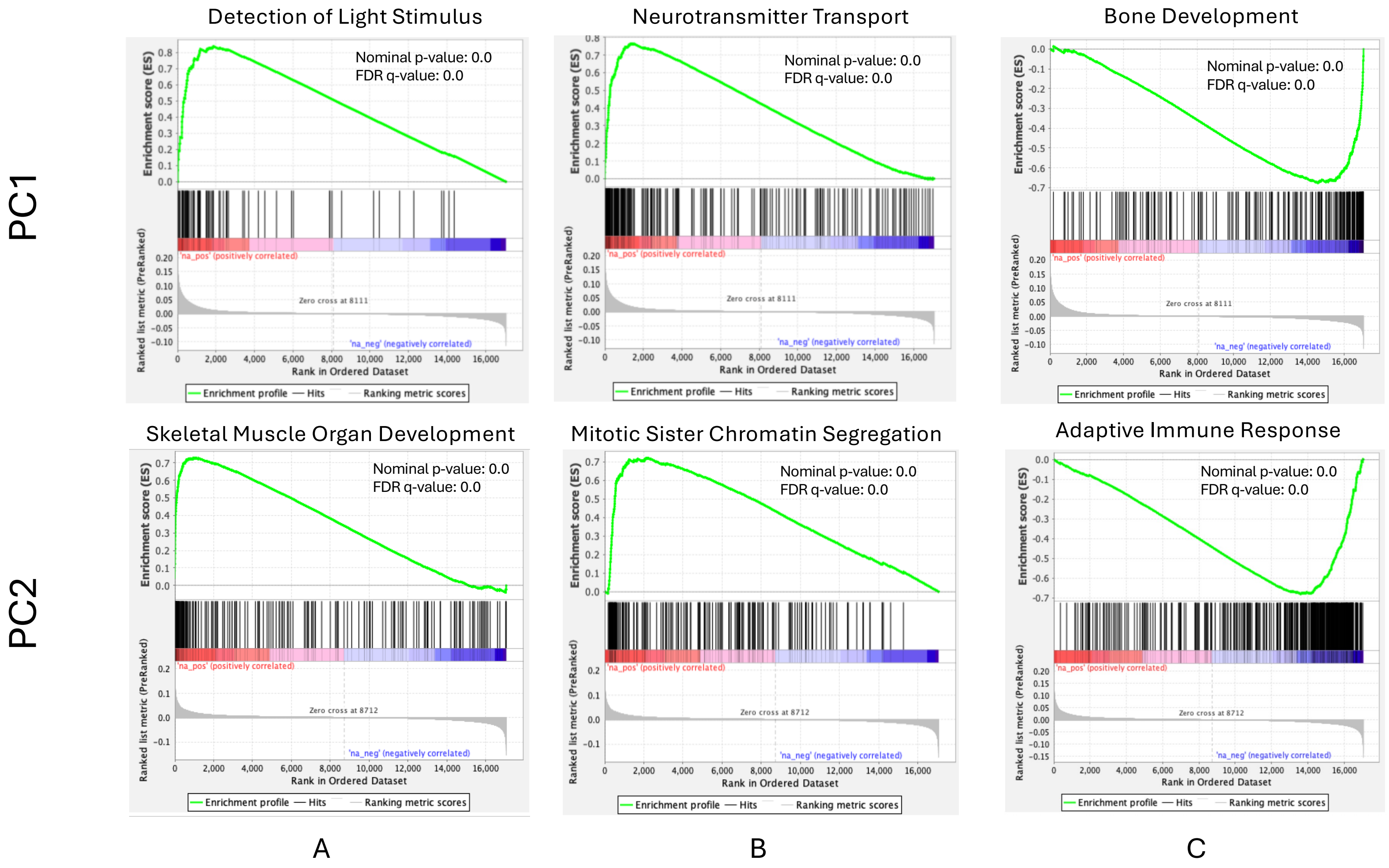

Figure S4 PC1 developmental and PC2 immune/myogenic signatures
Using the weighted loadings for Solid-Tumor PC1 and PC2 we performed GSEA to identify biological processes associated with genes driving these components. PC1 was enriched for developmental pathways, reflecting variation in tumor lineage and differentiation. PC2 showed immune-related signatures on the negative leading edge and mitotic and myogenic pathways on the positive leading edge. Panels A and B display enriched gene sets for up-weighted genes, while Panel C shows an enriched set for down-weighted genes.

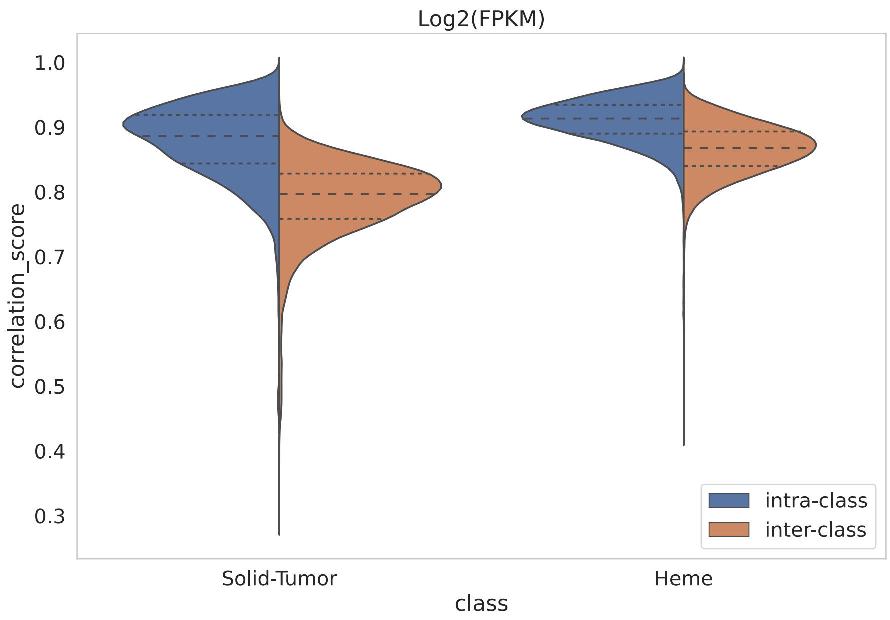

Figure S5 Hematologic dataset shows a narrower range of inter- and intra-class correlation scores

The solid tumor cohort exhibits an inter-type correlation of 0.788 ± 0.062 and an intra-type correlation of 0.876 ± 0.061, whereas the hematologic malignancy cohort demonstrates higher correlations, with an inter-type correlation of 0.865 ± 0.041 and an intra-type correlation of 0.910 ± 0.035.

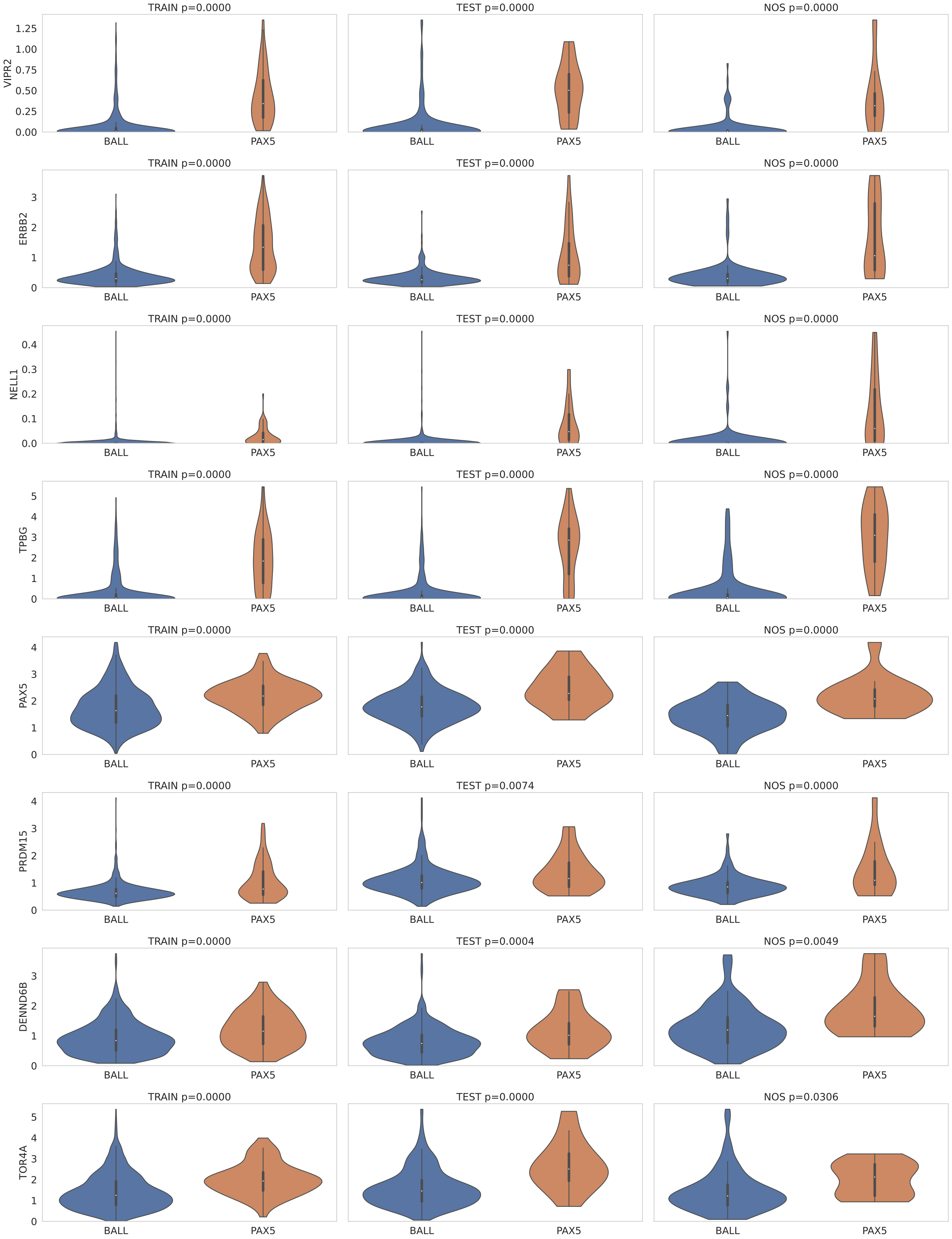

Figure S6 Expression patterns of candidate PAX5 subtype marker genes
PAX5, a key regulator of B cell development, is frequently disrupted in B-ALL, B cell acute lymphoblastic leukemia. Candidate *PAX5*-subtype marker genes were identified by combining the top overexpressed genes in the *PAX5* subtype versus other B-ALLs in the training set (*VIPR2*, *ERBB2*, *NELL1*, *TPBG*) with literature-reported genes (*PAX5*, *PRDM15*, *DENND6B*, *TORA*). CanID-predicted subtypes (*PAX5* vs B-ALL) in the NOS group mirrored the expression patterns observed in both the training and TestData1 sets.

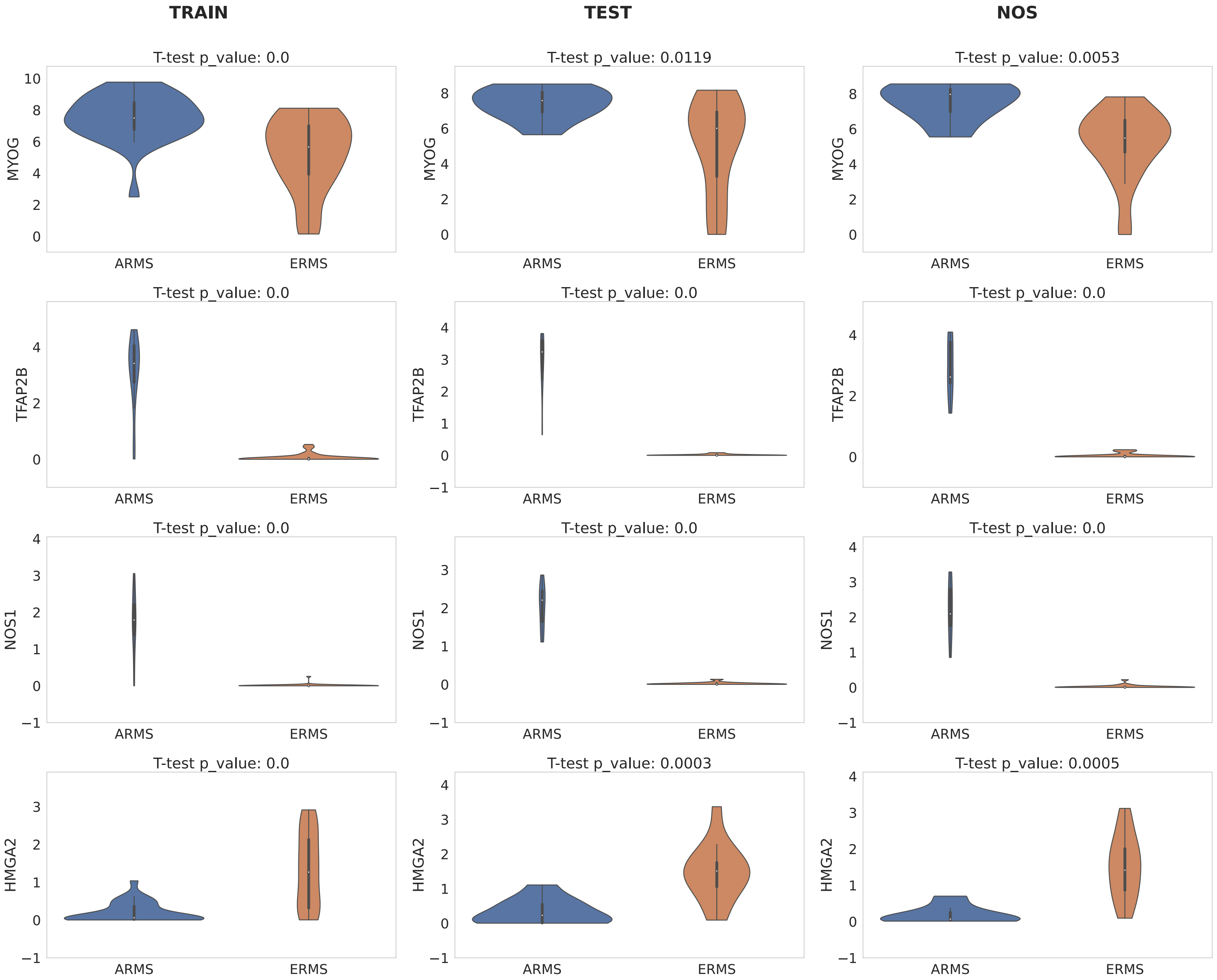

Figure S7 Expression pattern of rhabdomyosarcoma surrogate markers for fusion status

Violin plots of train, test, and NOS, not otherwise specified gene expression for *MYOG*, *TFAP2B*, *NOS1*, and *HMGA2*. *MYOG*, *TFAP2B*, and *NOS1* have higher expression in ARMS, alveolar rhabdomyosarcoma, while *HMGA2* has higher expression in ERMS, embryonal rhabdomyosarcoma. The CanID predicted subtype (ARMS vs ERMS) in the NOS column mirror the expression patterns seen in the train and TestData1 set.

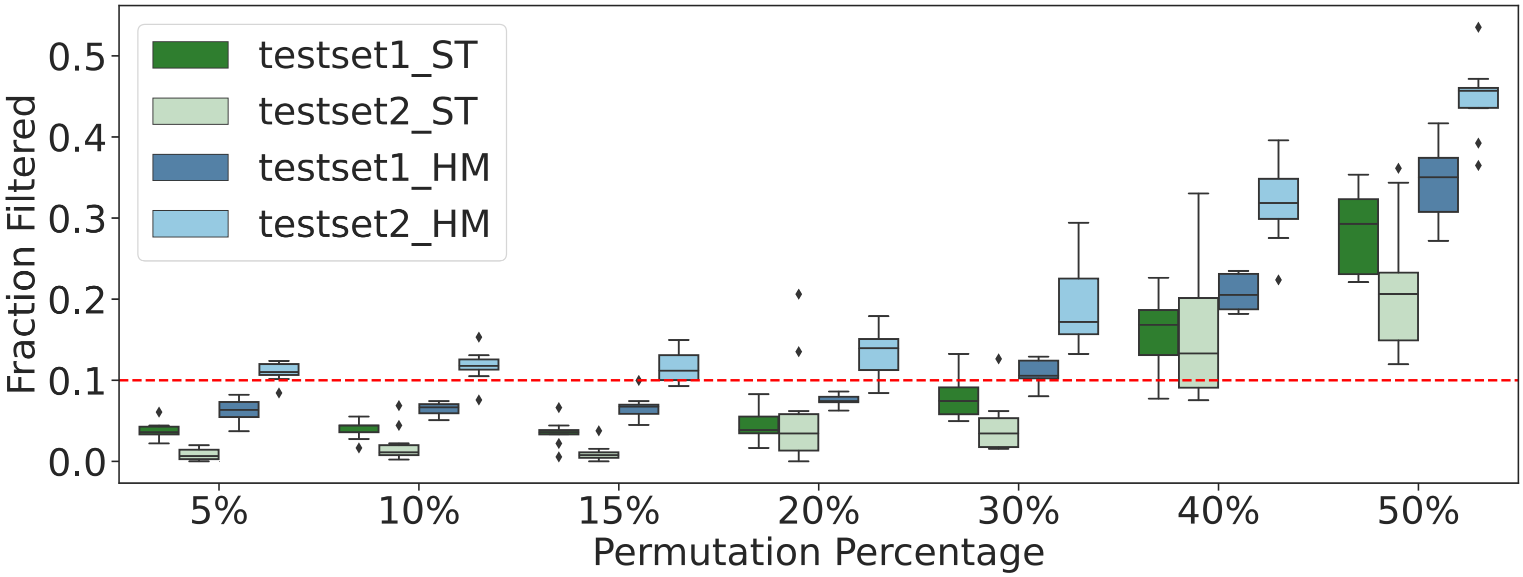

Figure S8 Performance robustness of solid tumor and hematologic malignancy CanID models under increasing sample label permutations

Overall performance on testset1 and testset2 is shown as the fraction of samples filtered across varying permutation percentages. The fraction filtered increases with higher permutation thresholds. The red dashed line marks a 10% filtering rate.

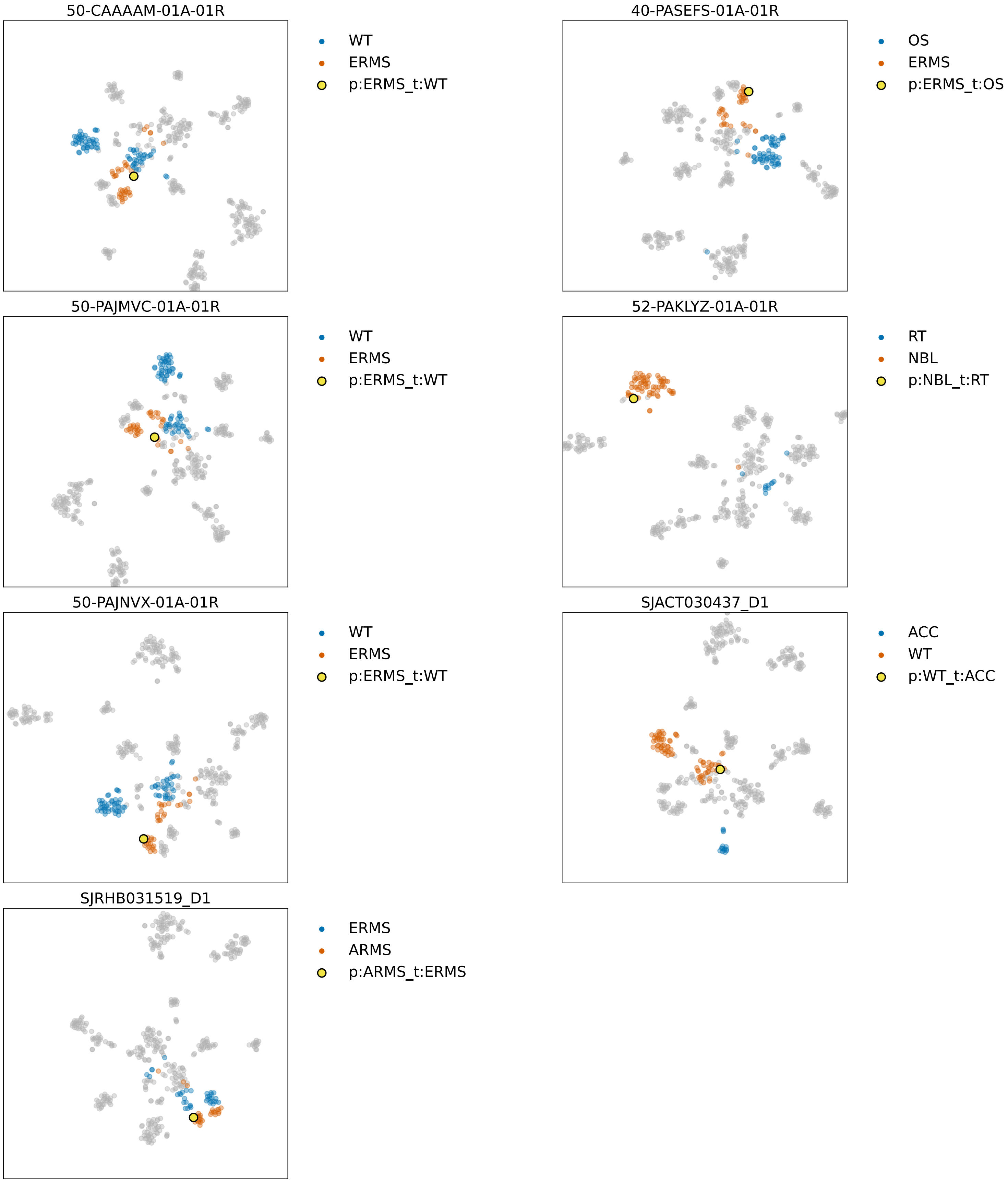

Figure S9 Plots of misclassified solid tumor samples cluster with their predicted type
Testset1: One ACC, adrenocortical carcinoma sample (SJACT030437_D1) was misclassified as WT, Wilms tumor and one ERMS, embryonal rhabdomyosarcoma sample (SJRHB031519_D1) was misclassified as ARMS, alveolar rhabdomyosarcoma. Testset2: Three WT samples (CAAAAM, PAJVMC, PAJNVX) were classified as ERMS, one OS, osteosarcoma sample (PASEFS), was classified as ERMS and one RT, rhabdoid tumor sample (PAKLYZ) was classified as NBL, neuroblastoma. The t-SNE projections were generated from raw counts converted to FPKM for the input gene set (N=17061).

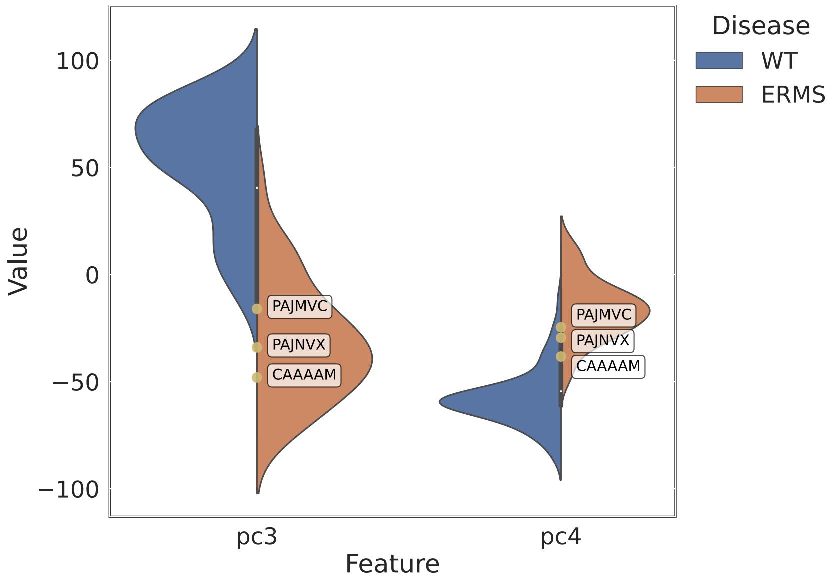

Figure S10 Misclassified Wilms tumor cases align with embryonal rhabdomyosarcoma profiles

Violin plots show the distributions of the principal components that distinguish ERMS, embryonal rhabdomyosarcoma (orange) from WT, Wilms tumor (blue) in the training data. Three TARGET, therapeutically applicable research to generate effective treatments samples—CAAAAM, PAJMVC, and PAJNVX—were labeled WT but predicted as ERMS and are shifted toward the ERMS distribution.

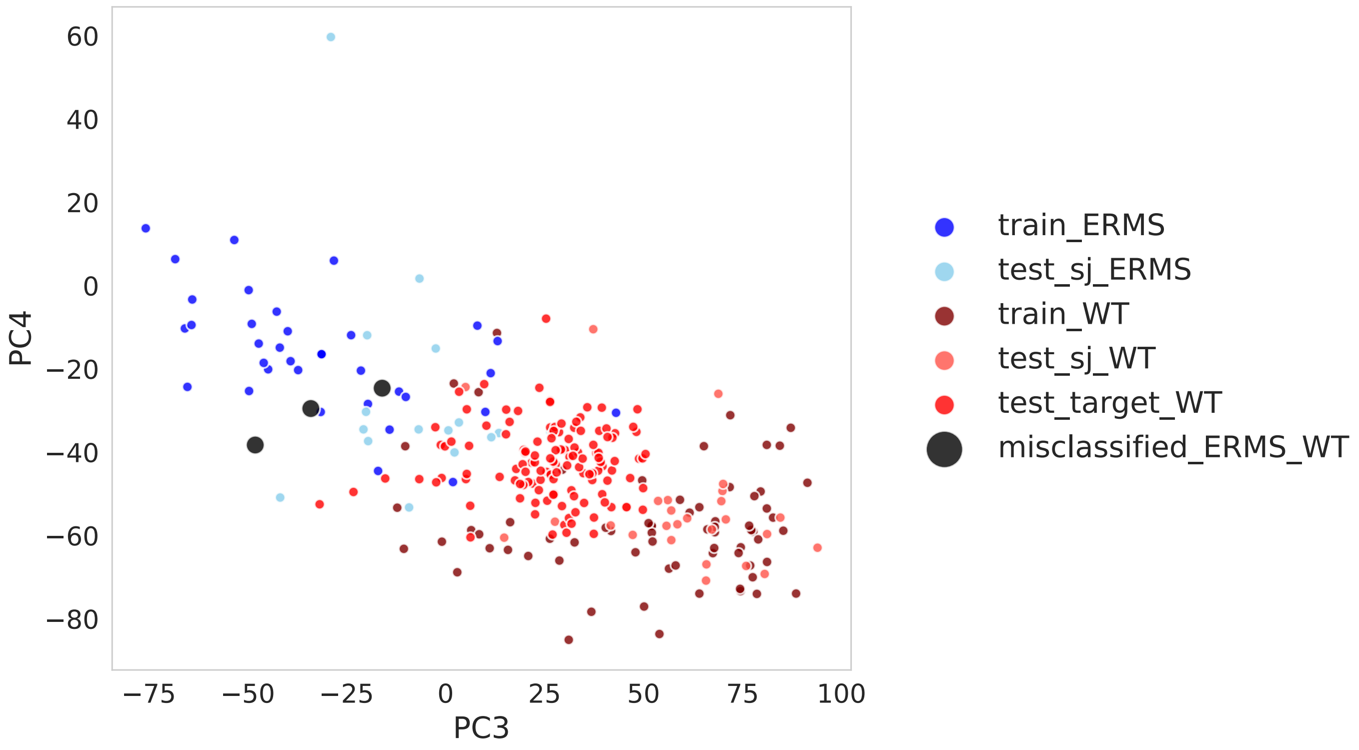

Figure S11 Principal component analysis highlights Wilms tumor cases misclassified as embryonal rhabdomyosarcoma

Samples shaded red denote WT, Wilms tumor; blue denotes ERMS, embryonal rhabdomyosarcoma. The three misclassified TARGET samples—CAAAAM, PAJMVC, and PAJNVX—labeled WT but predicted as ERMS, are shown as black points and cluster closer to the ERMS group than to WT.

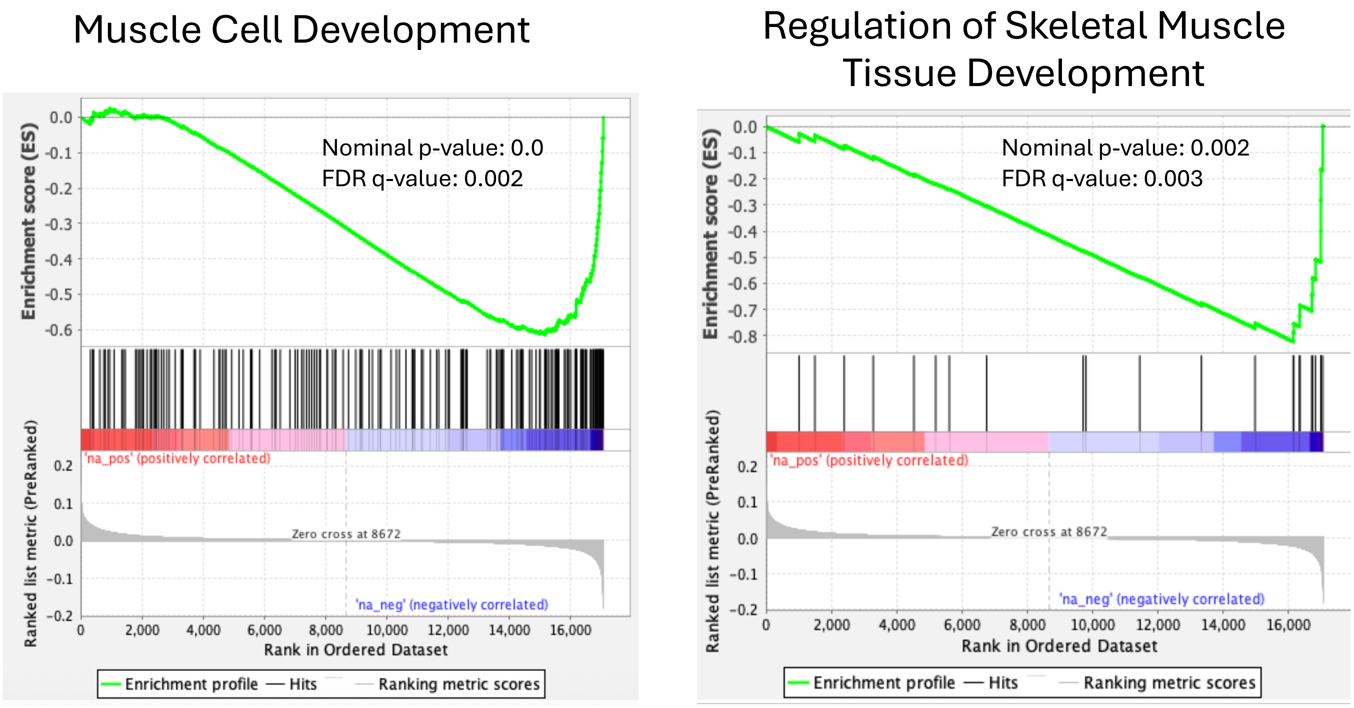

Figure S12 Principal component 3 captures myogenic differentiation programs
GSEA, gene set enrichment analysis show enrichment on the negative leading edge for pathways related to myogenic differentiation. This indicates that principal component 3 reflects variation driven by activation of muscle-lineage developmental programs in tumors.

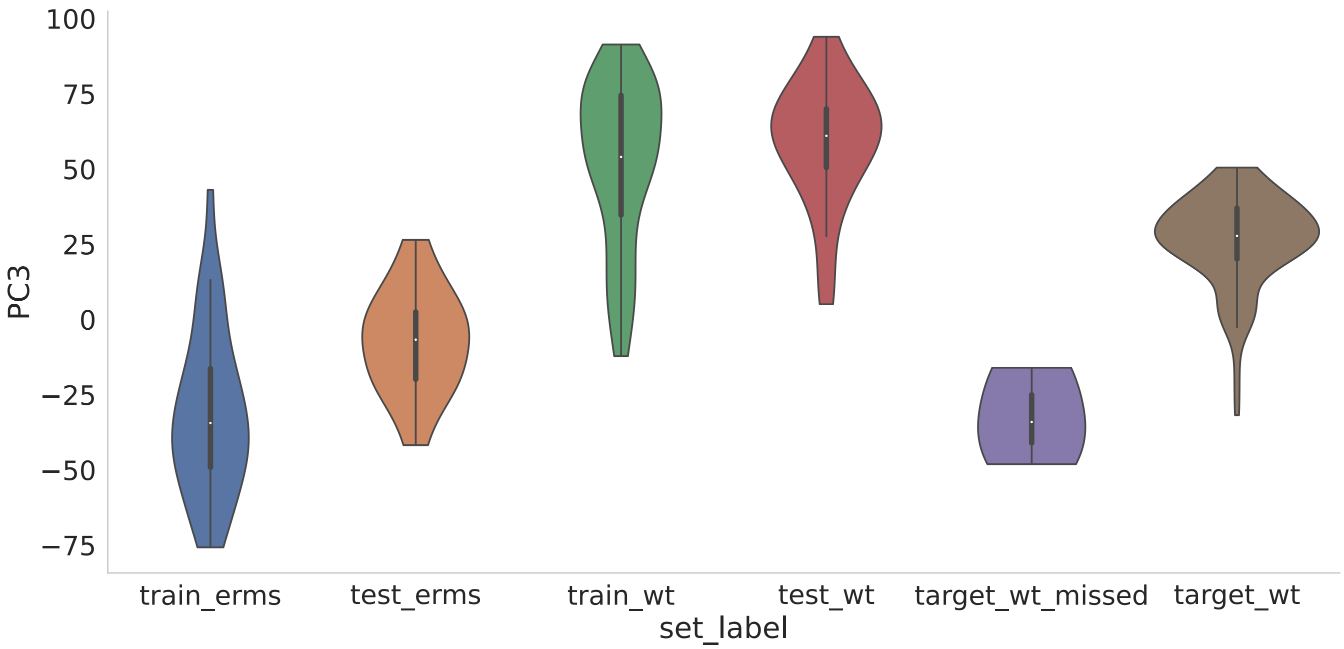

Figure S13 Principal component 3 distinguishes Wilms tumor from embryonal rhabdomyosarcoma

Violin plots of PC3, principal component values show that the three misclassified TARGET samples (CAAAAM, PAJMVC, PAJNVX) have negative PC3 values (−32.7 ± 13.1), aligning with embryonal rhabdomyosarcoma training samples (−24.0 ± 26.8) rather than Wilms tumor samples (38.6 ± 24.6).

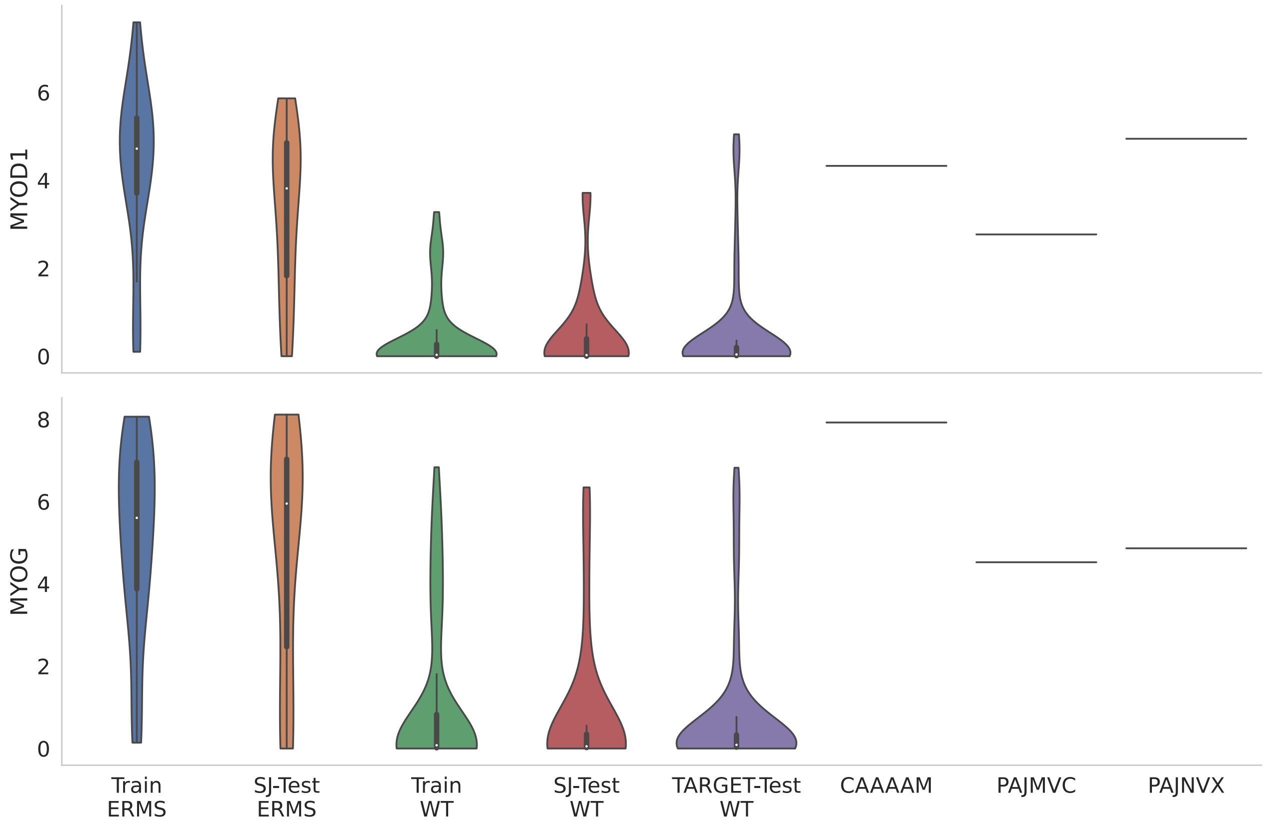

Figure S14 Elevated myogenic gene expression observed in Wilms tumor cases misclassified as embryonal rhabdomyosarcoma

Three Wilms tumor samples from the TARGET cohort—CAAAAM, PAJMVC, and PAJNVX—predicted as embryonal rhabdomyosarcoma show elevated *MYOD1* and *MYOG* expression, consistent with activation of myogenic differentiation programs. *MYOD1* and *MYOG* are master transcriptional regulators of myogenesis.

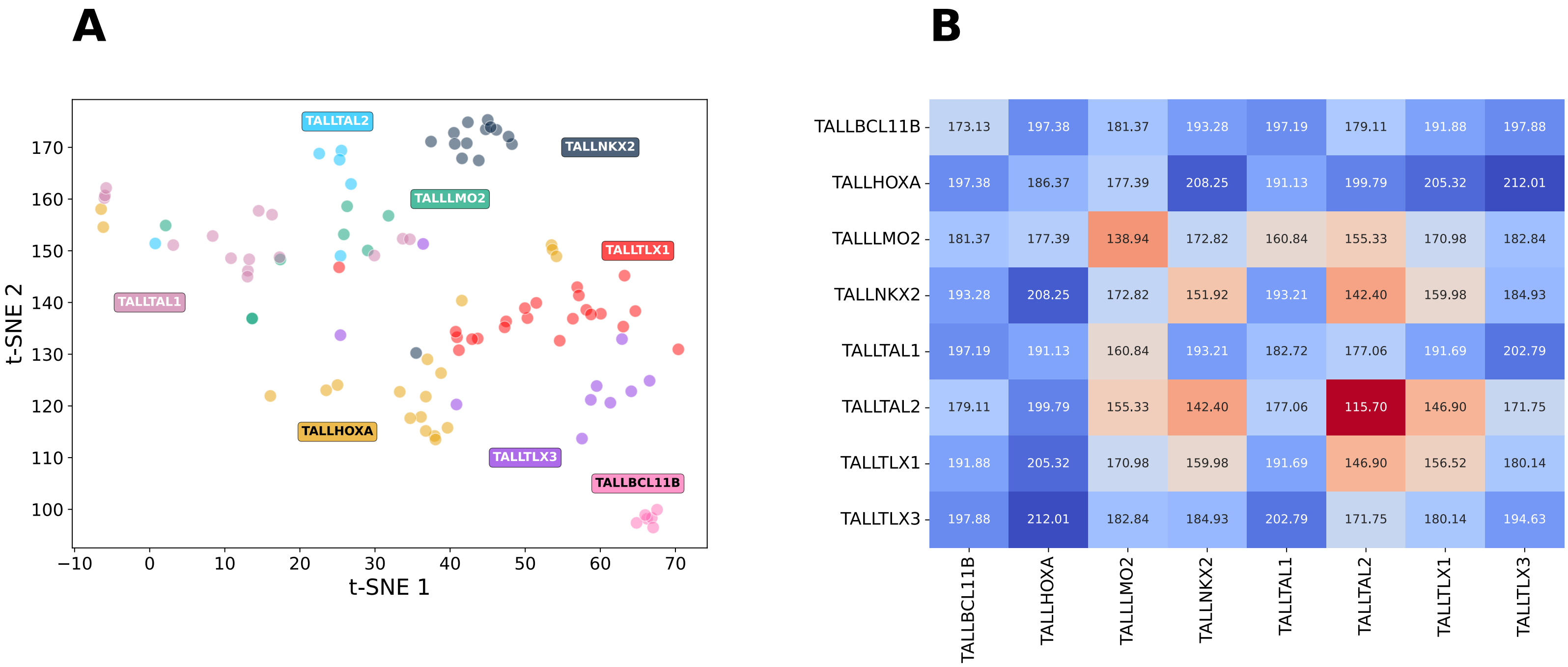

Figure S15 LMO2 T-cell acute lymphoblastic leukema cases align with the TAL1 subtype
A t-SNE, t stochastic neighborhood embedding projection of training T-ALL samples derived from principal component features (A) shows TALL-LMO2 tumor classes co-cluster with TALL-TAL1 tumor classes rather than forming a distinct group. A heat map of mean pairwise Euclidean distances among training expression profiles (B) indicates TAL1 is closest to LMO2: the mean TAL1–LMO2 distance is lower than the TAL1–TAL1 within-subtype distance and lower than distances to all other T-ALL subtypes.
